# Properties of Oligomeric Interaction of the Cytomegalovirus Core Nuclear Egress Complex (NEC) and Its Sensitivity to an NEC Inhibitory Small Molecule

**DOI:** 10.3390/v13030462

**Published:** 2021-03-11

**Authors:** Jintawee Kicuntod, Sewar Alkhashrom, Sigrun Häge, Benedikt Diewald, Regina Müller, Friedrich Hahn, Peter Lischka, Heinrich Sticht, Jutta Eichler, Manfred Marschall

**Affiliations:** 1 Institute for Clinical and Molecular Virology, Friedrich-Alexander University of Erlangen-Nürnberg (FAU), 91054 Erlangen, Germany; jintawee.kicuntod@extern.uk-erlangen.de (J.K.); sigrun.haege@fau.de (S.H.); mueller.regina@uk-erlangen.de (R.M.); friedrich.hahn@uk-erlangen.de (F.H.); 2 Department of Chemistry and Pharmacy, Division of Medicinal Chemistry, Friedrich-Alexander University of Erlangen-Nürnberg (FAU), Erlangen-Nürnberg, 91058 Erlangen, Germany; sewar.alkhashrom@fau.de (S.A.); jutta.eichler@fau.de (J.E.); 3 Division of Bioinformatics, Institute of Biochemistry, Friedrich-Alexander University of Erlangen-Nürnberg (FAU), Erlangen-Nürnberg, 91054 Erlangen, Germany; benedikt@diewald.com (B.D.); heinrich.sticht@fau.de (H.S.); 4 AiCuris Anti-Infective Cures GmbH, 42117 Wuppertal, Germany; peter.lischka@aicuris.com

**Keywords:** human cytomegalovirus, core nuclear egress complex (NEC), in vitro NEC assembly assay, core NEC coimmunoprecipitation, oligomeric interaction properties, associated cellular factors, NEC-blocking small molecule, antiviral targeting strategy

## Abstract

Herpesviral nuclear egress is a regulated process shared by all family members, ensuring the efficient cytoplasmic release of viral capsids. In the case of human cytomegalovirus (HCMV), the core of the nuclear egress complex (NEC) consists of the pUL50-pUL53 heterodimer that builds hexameric lattices for capsid binding and multicomponent interaction, including NEC-associated host factors. A characteristic feature of NEC interaction is the N-terminal hook structure of pUL53 that binds to an alpha-helical groove of pUL50, thus termed as hook-into-groove interaction. This central regulatory element is essential for viral replication and shows structural–functional conservation, which has been postulated as a next-generation target of antiviral strategies. However, a solid validation of this concept has been missing. In the present study, we focused on the properties of oligomeric HCMV core NEC interaction and the antiviral activity of specifically targeted prototype inhibitors. Our data suggest the following: (i) transiently expressed, variably tagged versions of HCMV NEC proteins exert hook-into-groove complexes, putatively in oligomeric assemblies that are distinguishable from heterodimers, as shown by in vitro assembly and coimmunoprecipitation approaches; (ii) this postulated oligomeric binding pattern was further supported by the use of a pUL50::pUL53 fusion construct also showing a pronounced multi-interaction potency; (iii) using confocal imaging cellular NEC-associated proteins were found partly colocalized with the tagged core NECs; (iv) a small inhibitory molecule, recently identified by an in vitro binding inhibition assay, was likewise active in blocking pUL50–pUL53 oligomeric assembly and in exerting antiviral activity in HCMV-infected fibroblasts. In summary, the findings refine the previous concept of HCMV core NEC formation and nominate this drug-accessible complex as a validated antiviral drug target.

## 1. Introduction

Human cytomegalovirus (HCMV) is a herpesvirus with a double-stranded DNA genome, categorized into subfamily *Betaherpesvirinae*. HCMV represents a dominant human pathogen comprising worldwide distribution. In immunocompetent individuals, the pathogenesis of infection by HCMV mostly exhibits a scale of clinically minor severity, from asymptomatic to mild forms of symptoms. However, the infection in immunocompromised, immunosuppressed and immunonaïve hosts can lead to severe systemic diseases and life-threatening complications. Notably, the congenital infection of HCMV raises the most concern since it may cover a broad range of symptoms as well as high morbidity and mortality in the unborn and infants. Generally, HCMV pathogenesis is determined by distinct factors of virus–host interaction, viral productivity, viremia, immune control and tissue tropism [1,2,3].

Regulation of the viral replication cycle is mediated by the complex interplay between viral and host proteins in a multifaceted way, including the formation of viral–cellular multiprotein complexes. In particular, the multicomponent HCMV-specific nuclear egress complex (NEC) exerts a rate-limiting step of productive replication and recently attracted high interest as it represents a putative target for novel antiviral strategies. As is the case for almost all DNA viruses, genomic replication of HCMV occurs in the host cell nucleus, where capsids are assembled, genome-packaged and exported to the cytoplasm for further maturation. A crucial step of the HCMV nucleo-cytoplasmic egress relies on the correct and efficient NEC formation, as primarily mediated by two essential viral egress proteins, pUL50 and pUL53, forming a heterodimer and serving as core NEC for further recruitment of effector proteins and viral capsids. In the case of HCMV, the recruited viral and cellular proteins have been identified through a variety of experimental settings [4,5]. Their fine-regulated assembly leads to the formation of the multicomponent NEC that includes the HCMV-encoded protein kinase pUL97, the multi-ligand binding protein p32/gC1qR, emerin, cellular kinases such as protein kinase C (PKC) and cyclin-dependent kinase 1 (CDK1), the prolyl cis/trans-isomerase Pin1 as well as a number of additional proteins [4,6]. Specifically, the multicomponent NEC stimulates a phosphorylation-triggered disruption of the nuclear lamina that facilitates the transition of viral capsids across the nuclear envelope.

In previous studies, the 3D crystal structure of the globular domain of the HCMV core NEC was resolved, also revealing a higher-order assembly of the pUL50–pUL53 heterodimers in the form of hexameric ring-like structures [7,8,9], which were similarly detected for other herpesviruses [10,11,12,13,14]. The 3D structural image of the HCMV core NEC strongly suggested a binding principle, termed hook-into-groove interaction since it is based on an N-terminal hook-like pUL53 protrusion that embraces an α-helical pUL50 binding groove. Functional analyses, performed with NEC proteins of α-, β- and γ-herpesviruses supported this concept [15,16,17,18,19,20,21,22,23,24,25,26,27]. Notably, the oligomeric ring-like structure of the HCMV core NEC may exquisitely serve as a docking platform that binds to the hexagonal surface structure of nuclear viral capsids [4] and thus may promote the directed capsid egress through the distorted nuclear lamina [28]. Seen from this background of scientific evidence, the pharmaceutical interference with viral NEC formation, and specifically with the dimeric and hexameric interaction between pUL50 and pUL53, provides a highly attractive target for novel antiviral strategies.

In this study, we focused on the establishment of test systems for the analysis of oligomeric HCMV core NEC interaction. A valuable model was provided by the transient expression of variably tagged versions of the HCMV NEC proteins to study their oligomeric interaction properties in in vitro assembly and coimmunoprecipitation approaches. Building on this, the sensitivity of NEC assemblies to a newly identified NEC inhibitory small molecule was demonstrated and the antiviral characteristics of this inhibitor are presented in this report and in a second, thematically closely linked report (co-submitted to be published in the same issue).

## 2. Materials and Methods

### 2.1. Cell Culture

Human embryonic kidney epithelial cells (HEK 293T, CRL-3216, ATCC) and HeLa cells (ATCC) were cultivated in Dulbecco’s modified Eagle medium (DMEM, 11960044, Thermo Fisher Scientific, Waltham, MA, USA). Primary human foreskin fibroblasts (HFFs), MRC-5 and ARPE-19 cells were cultivated in minimal essential medium (MEM). Cell culture media were supplemented with 1× GlutaMAX™ (35050038, Thermo Fisher Scientific), 10 μg/mL gentamicin and 10% fetal bovine serum (FBS, F7524, Sigma Aldrich, St. Louis, MO, USA). All cells were incubated at 37 °C, 5% CO_2_ and 80% humidity.

### 2.2. Plasmid Constructs

Expression plasmids coding for several tags (HA, AU1, Flag and His) of HCMV pUL50 or pUL53 were generated by PCR amplification of the UL50 or UL53 open reading frame (ORF), using the template pCM1029 [29]. In addition to individual full-length UL50 or UL53, a fusion version (UL50::UL53), encoding aa 1-397 of UL50 and aa 1-376 of UL53, was also generated. Primers containing tag sequences were also amplified via PCR, resulted in the fusion of the ORFs to C-terminal with different tags. Vent DNA polymerase (New England Biolabs, Ipswich, MA, USA) was obtained for performing PCR with 36 cycles (denaturation for 40 s at 94 °C, annealing for 40 s at 58 °C and polymerization for 90 s at 72 °C). PCR products were cleaved with the restriction enzymes EcoRI/XhoI and were inserted into the vector pcDNA3.1 (Invitrogen). The plasmids coding of HCMV pUL50 or pUL53 expressing red (RFP) or green fluorescent protein (GFP) were inserted into vectors pDsRed1-N1 or pEGFP-N1 (both BD Clontech), respectively after cleavage with the restriction enzyme EcoRI/BamHI. Oligonucleotide primers used for PCR were purchased from Biomers (Supplementary Materials Table S1).

### 2.3. Antiviral Compounds

Compounds were derived from the commercially available Prestwick Chemical Library^®^ (Prestwick Chemical company, Illkirch-Graffenstaden, France, prestwickchemical.com) and utilized for a screening based on a pUL50–pUL53 in vitro binding assay described elsewhere [30]. The identified hit compound merbromin (MBM) was purchased from Sigma-Aldrich (M7011). Reference drugs were used as follows, ganciclovir (GCV; Sigma-Aldrich), maribavir (MBV; Shanghai PI Chemicals Ltd., Shanghai, China) and PiB (Sigma-Aldrich). All drugs were dissolved in DMSO stock solutions and stored at −20 °C.

### 2.4. Antibodies

Monoclonal (mAb) and polyclonal (pAb) antibodies were used to detect the following cellular proteins: mouse mAb-emerin (H-12, Santa Cruz Biotech, Dallas, TX, USA), mouse mAb-p32 (anti-p32/gC1qR, Abcam, Cambridge, UK), mouse mAb-CDK1 (MA5-11472, Thermo Fisher), rabbit pAb-CDK1 (Sc-954, Santa Cruz Biotech), mouse mAb-PKCα (A-3, Santa Cruz Biotech), rabbit mAb-lamin A/C (EPR4100, Abcam) and mouse mAb-β-actin (AC-15, Sigma Aldrich). Mouse mAb-Flag (M2-1804, Sigma-Aldrich), mouse mAb-HA (clone HA-7, Sigma-Aldrich), mouse mAb-His (MA1-21315, Thermo Fisher) and mouse mAb-AU1 (MMS130P, Biolegend, San Diego, CA, USA) were used for coimmunoprecipitation settings (CoIP) and mouse IgG F_C_ (Dianova, Hamburg, Germany) was used as a CoIP negative control. Additional antibodies against transiently expressed tagged proteins were used as follows: mouse mAb-His (MA1-21315, Thermo Fisher), rabbit pAb-HA (T501, Signalway Antibody, College Park, MD, USA), mouse mAb-GFP (11814460001, Roche), rabbit pAb-RFP (NBP1-69962, Novus, Wiesbaden, Germany), goat pAb-AU1-HRP (PA1-26549, Invitrogen Thermo Fisher), mouse mAb-Flag-HRP (Sigma Aldrich) and rat mAb-HA-HRP (3F10, Roche, Basel, Switzerland). HCMV-specific antibodies were used as follows: rabbit pAb-IE86, mouse mAb-IE1p72 (P63-27), mouse mAb-pp150 (36-14/XPA), mouse mAb-UL50.01 and mouse mAb-UL53.01 (kindly provided by Prof. Stipan Jonjic and Dr. Tihana Lenac Rovis, University of Rijeka, Rijeka, Croatia). Alexa Fluor 488-, 555- and 647-conjugated antibodies were employed as secondary antibodies for indirect immunofluorescence staining (Molecular Probes, Eugene, OR, USA) and horseradish peroxidase-conjugated anti-mouse and -rabbit (Dianova, Hamburg, Germany) for Western blot analysis.

### 2.5. PEI Transfection and Coimmunoprecipitation (CoIP)

293T cells were seeded in 10 cm Petri dishes with a density of 5 × 10^6^ cells per dish and incubated at 37 °C overnight prior to transfection. After that, 293T cells were transiently transfected with combined plasmids of pcDNA-UL50 and pcDNA-UL53 in different tags and/or with pcDNA-UL50::UL53-AU1 by polyethylenimine (PEI)-DNA complexes (Sigma Aldrich) as described previously [31]. Two to three days after incubation at 37 °C, transiently transfected 293T cells were harvested and assessed for protein–protein interaction via CoIP assay. The Dynabeads™ Protein A (10002D, Thermo Fisher Scientific) were coupled with tag-specific antibodies for immunoprecipitation. Cell lysates that contained the plasmids coding for proteins of pcDNA-UL50 and pcDNA-UL53 in various tags were added to Dynabeads™ Protein A and continued to incubate at 4 °C for approx. 4 h. Then, unbound proteins were washed out and the CoIP samples were analyzed by Western blot (Wb).

### 2.6. In Vitro NEC Assembly Assay

293T cells with a density of 5 × 10^6^ cells were cultivated in each 10 cm petri dish. After incubation at 37 °C overnight, 293T cells were transiently transfected with plasmids by PEI transfection. Transfected cells were harvested 2–3 days post-transfection (d p.t.) for in vitro assembly-based CoIP. Cell pellets were resuspended in 600 µL CoIP buffer (1 M Tris/HCl pH 8.0, 5 M NaCl, 0.5 M EDTA and 10% NP40) including proteolytic inhibitors such as 20 µM phenylmethylsulfonyl fluoride (PMSF). Lysis was achieved by sonication on ice at 80% of duty cycles for at least 20 s and continued to lyse on ice for 20 min. The suspensions were then centrifuged for 10 min at 14,000 rpm at 4 °C to remove insoluble debris, and thereafter 100 µL of the different homogeneous lysates were combined for protein assembly in 1.5-mL microcentrifuge tubes and vector lysate was used to equalize identical volumes. The assembly reactions were incubated at 4 °C overnight under rotation. Thereafter, the assembled protein complexes were incubated with antibody-coated Dynabeads™ Protein A (antibody concentrations according to manufacturers’ information) at 4 °C for approximately 4 h. Finally, the beads were washed to remove the unbound protein fraction, before CoIP samples were analyzed by Wb to detect protein-protein interactions. Optionally, inhibitors were added to the combined lysates for the determination of direct NEC-inhibiting effects.

### 2.7. Virus Infection

Viral stocks of HCMV strains AD169, TB40E, TB40 UL32-GFP and Merlin were produced on HFFs or ARPE-19 (TB40E), respectively, and infectious titers were determined by the plaque assay. For the generation of samples to be used for confocal imaging, 2.5 × 10^5^ HFFs, MRC-5 or ARPE-19 cells were cultivated in 6-well plates on coverslip at 1 d prior to infection with the indicated strains of HCMV. After 90 min of viral adsorption, the inocula were removed, media were refreshed and cells were further cultivated at 37 °C. Under conditions of inhibitor treatment, cells were incubated with the indicated concentrations of compounds in the culture media at 37 °C for 5 d, before cells were fixed and subjected to indirect immunofluorescence staining and evaluation by confocal laser-scanning microscopy.

### 2.8. FuGENE^®^ Transient Transfection, Indirect Immunofluorescence Assay and Confocal Laser-Scanning Microscopy

HeLa cells were grown on coverslips at a density of 3.5 × 10^5^ cells in 6-well plates and incubated at 37 °C overnight. The next day, cells were transiently transfected with pcDNA3.1, pcDNA-UL50-HA, pEGFP-UL53 and pcDNA-UL50::UL53-AU1 by FuGENE^®^ HD transfection reagent according to the manufacturer’s instructions. Transfected cells were incubated at 37 °C for 2 d prior to performing indirect immunofluorescence staining. For this purpose, transiently transfected HeLa cells or HCMV-infected HFFs, MRC-5 or ARPE-19 cells were fixed and permeabilized at the indicated time points and were stained under conditions previously described [29,32]. A TCS SP5 confocal laser-scanning microscope (Leica Microsystems, Wetzlar, Germany) was used for collecting the images. Images of a confocal plane were taken with a pinhole of 1 airy unit and a line average of 3 and were analyzed by LAS AF software (Leica Microsystems, Wetzlar, Germany). Visual microscopic counting was performed for quantitative evaluations. Student’s *t*-test was applied to determine statistical significance.

### 2.9. Bioinformatic Methods

The conformational stability of pUL50 was investigated by molecular dynamics (MD) simulations using the program AMBER14 [33]. The setup of the MD simulations was identical to that described in [16]. Two independent simulations of 300 ns length were performed starting from the pUL50 conformation observed in the pUL50–pUL53 complex crystal structure (PDB: 5D5N; [9]). Structural comparison to MCMV pM50 was based on the NMR structure of pM50 (PDB: 5A3G; [34]). The program Chimera [35] was used for structural analysis and visualization.

## 3. Results

### 3.1. Functional Principle of the HCMV-Specific Nuclear Egress Complex and Generation of Test Constructs to Analyze Its Oligomeric Binding Properties

**Figure 1:**
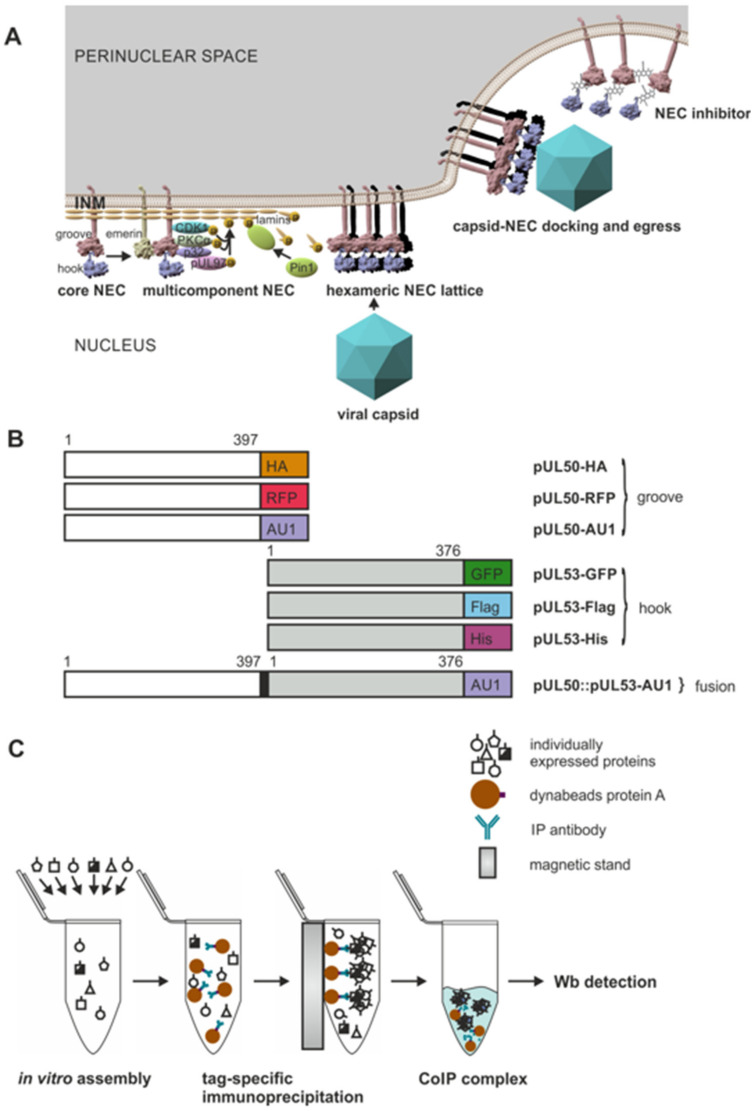
Schematic illustration of the functional role and approaches for analysis of the human cytomegalovirus (HCMV) core nuclear egress complex (NEC). (**A**) The core of the HCMV-specific NEC is built by the pUL50–pUL53 complex (termed as groove and hook proteins, respectively) that recruits effector proteins of the multicomponent NEC, including the viral protein kinase pUL97, the bridging factor p32/gC1qR, emerin, protein kinase C (PKC)α, cyclin-dependent kinase 1 (CDK1) and the peptidyl-prolyl *cis*/*trans* isomerase Pin1. Moreover, hexameric arrangements of pUL50–pUL53 form a receptor lattice for the intranuclear docking of maturing progeny capsids, ultimately guiding them to inner nuclear membrane (INM) budding for perinuclear egress. At present, it is not known yet whether the HCMV-specific formation of a multicomponent NEC and a hexameric core NEC lattice are two processes taking place simultaneously or whether one of these depends on the other. Note, however, that this sophisticated functional interlocking of events, particularly the step of oligomeric pUL50–pUL53 assembly, has been considered as a sensitive target for NEC inhibitory small molecules. (**B**) A series of differentially tagged expression constructs were generated for the analysis of oligomeric interaction, including constructs for groove, hook and pUL50::pUL53 fusion proteins. (**C**) These constructs were used for the single transient expression of tagged versions of pUL50 and pUL53, to be analyzed in a newly established oligomeric NEC in vitro assembly assay. Oligomer formation could be demonstrated by coimmunoprecipitation and Western blot detection using the respective tag-specific antibodies.

Recent investigations corroborated the central role of the viral core proteins pUL50 and pUL53 in the formation of the HCMV-specific NEC, as reviewed earlier ([4,22] and references therein) and summarized by Figure 1A. The molecular mode of pUL50–pUL53 binding has been termed ‘hook-into-groove interaction’ based on the unique α-helical structures of binding interfaces [9]. As another crucial feature, the pUL50–pUL53 core complex of the NEC is able to undergo hexameric arrangements, as identified in crystal structures of recombinant proteins [7,8,9] and supported by cryo-electron microscopy for related NECs from large unilamellar vesicles [11]. Here, we addressed the question of whether the property of oligomeric assembly of pUL50–pUL53 may likewise be detectable in solution when using protein lysates from transiently transfected cells. To this end, a series of differentially tagged expression constructs were generated for both pUL50 and pUL53 (Figure 1B). Using total lysates of 293T cells transiently transfected with either of these constructs, an in vitro assembly assay was established to analyze the oligomeric NEC assembly (Figure 1C), based on conditions reported earlier for the in vitro assembly of pUL97-cyclin [36] or related herpesviral NEC interactions [18].

### 3.2. An In Vitro NEC Assembly Assay Strongly Supports the Idea of Oligomeric HCMV pUL50-pUL53 Interaction

**Figure 2:**
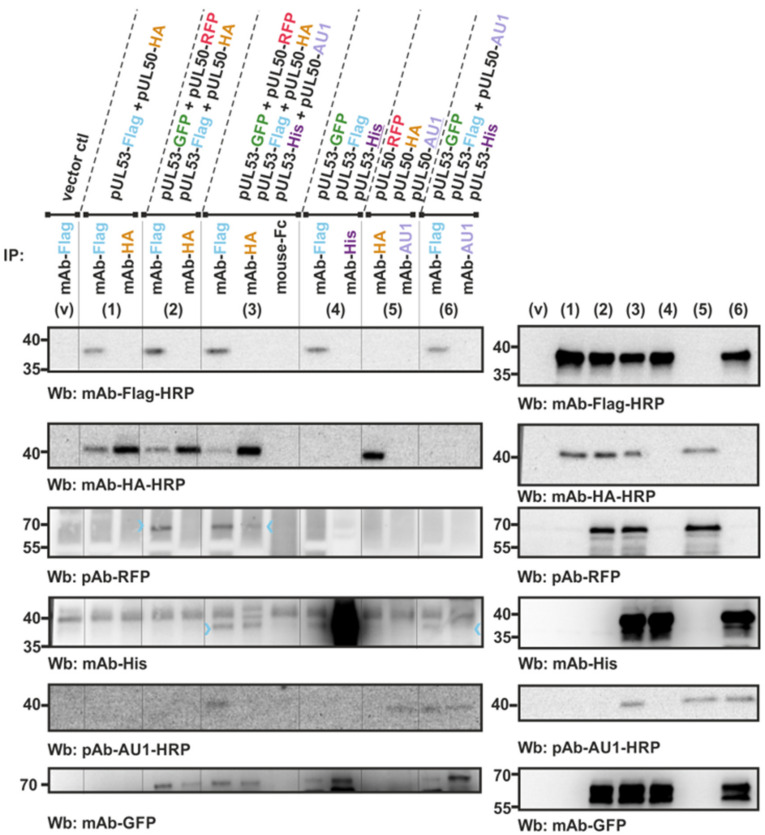
In vitro NEC assembly assay demonstrating the basic potential of oligomeric interaction of the HCMV core NEC proteins pUL50 (groove) and pUL53 (hook). The indicated expression plasmids, as described by Figure 1B, were used for the single transient transfection of 293T cells and harvested at approx. 2 days post-transfection (d p.t.) for the preparation of total lysates. Expression levels of the individual proteins were monitored by the detection of lysate controls on Western blots (right panels: v, vector control; 1–6, single-expression protein samples, subsequently taken as the input for in vitro assembly reactions, as described by Figure 1C). Several combinations of these samples were used in twofold, threefold, fourfold or sixfold settings of in vitro assembly as indicated above the immunoblots (left panels: v, vector control; 1–6, combinations of in vitro assembly reactions; incubated at 4 °C overnight under rotation). Thereafter, the assembled complexes were subjected to coimmunoprecipitation settings (CoIP), using the indicated immunoprecipitation antibodies (IP), and subsequently analyzed in a standard SDS-PAGE/Wb procedure, using the indicated antibodies for immunostaining (Wb). Colored arrowheads indicate weakly detectable bands.

Using the generated test constructs under appropriate conditions of the in vitro assembly, the detectability of oligomeric interaction patterns was addressed. Actually, by performing a pUL50–pUL53 in vitro assembly using several different combinations of tagged protein versions, the specific oligomeric interaction properties of the two viral proteins were demonstrated (Figure 2). First, the Western blot detection of the transiently expressed proteins ensured the presence of sufficient amounts of individually tagged versions of the NEC proteins (Figure 2, right panels), which were then used as the input for CoIP analysis (Figure 2, left panels). A sample from vector-transfected cells (V) served as a negative control monitoring the lack of nonspecific binding or cross-reactivity in the immuno-detection. Then, the positive control of pUL50–pUL53 interaction was performed with the twofold combination between pUL53-Flag and pUL50-HA (Figure 2, panel 1). Immunoprecipitation was positive for both antibodies (immunoprecipitation antibodies (IP)-Ab), mAb-Flag and mAb-HA, but a strong CoIP signal was mostly restricted to the Flag-specific IP. When analyzing fourfold (panel 2) or sixfold combinations (panel 3) of mixed pUL53/pUL50 settings, the tag-specific detection of CoIP signals obtained for an IP with mAb-Flag indicated the positive interaction patterns of all four or six involved protein versions, respectively (Figure 2, panels 2–3, mAb-Flag as IP-Ab, colored arrowheads indicate weakly detectable bands). It should be mentioned that in panel 3, the experimental setting was not able to distinguish between a true hexameric arrangement or the presence of various lower-order complexes. Nevertheless, the combined results strongly suggest a pattern of more-than-binary binding and are compatible with the concept of oligomeric core NEC interaction even in the absence of additional viral proteins. Interestingly, the exclusive presence of either three hook pUL53 versions alone (panel 4) or three groove pUL50 versions alone (panel 5) did not result in the detectable formation of higher-order complexes. In the case of the addition of one groove-like version (pUL50-AU1) to three hook pUL53 versions, however, led to a slightly detectable interaction of the four components (Figure 2, panel 6, mAb-Flag as IP-Ab, colored arrowhead indicates weakly detectable band). In conclusion, the approach of in vitro assembly-based CoIP provided data that strengthened our earlier postulate of the oligomeric assembly properties of the HCMV core NEC.

### 3.3. Confirmation of Conditions of Oligomeric pUL50-pUL53 Interaction Using the Standard CoIP Approach

**Figure 3:**
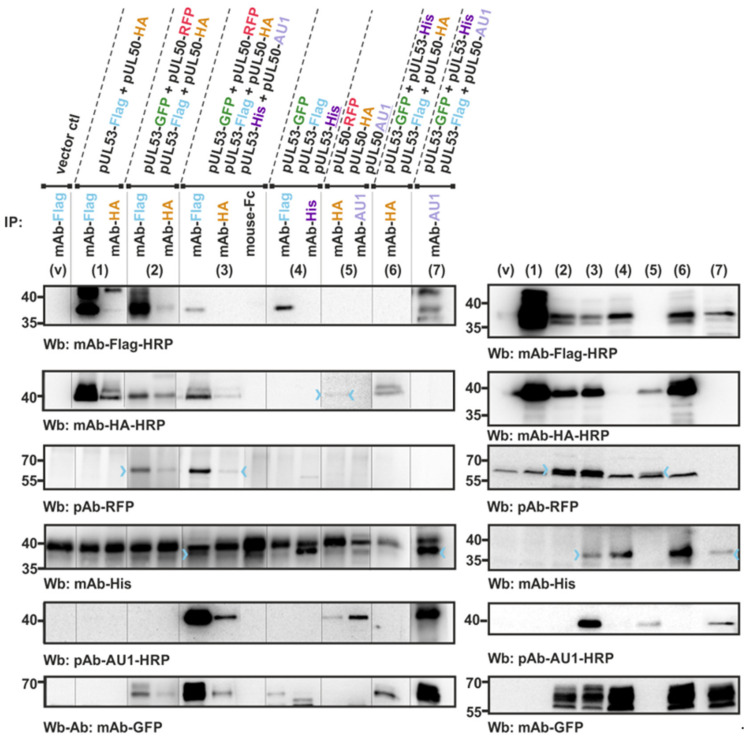
Cotransfection-based CoIP analysis as a confirmation test supporting the oligomeric interaction properties of the HCMV core NEC proteins pUL50 (groove) and pUL53 (hook). The indicated expression plasmids were used for the transient cotransfection of 293T cells and harvested at approx. 2 d p.t. for the preparation of total lysates. Expression levels of the individual proteins were monitored by the detection of lysate controls on Western blots (right panels: v, vector control; 1–6, coexpression protein samples, directly taken as the input for standard CoIP). The combinations of twofold, threefold, fourfold or sixfold coexpression is indicated above the immunoblots (left panels: v, vector control; 1–6, coexpression lysates used for CoIP; incubated at 4 °C for approx. 4 h under rotation). Thereafter, the CoIP samples were washed and subsequently analyzed in a standard SDS-PAGE/Wb procedure, using the indicated antibodies for immunostaining (Wb). Colored arrowheads indicate weakly detectable bands.

**Figure 4:**
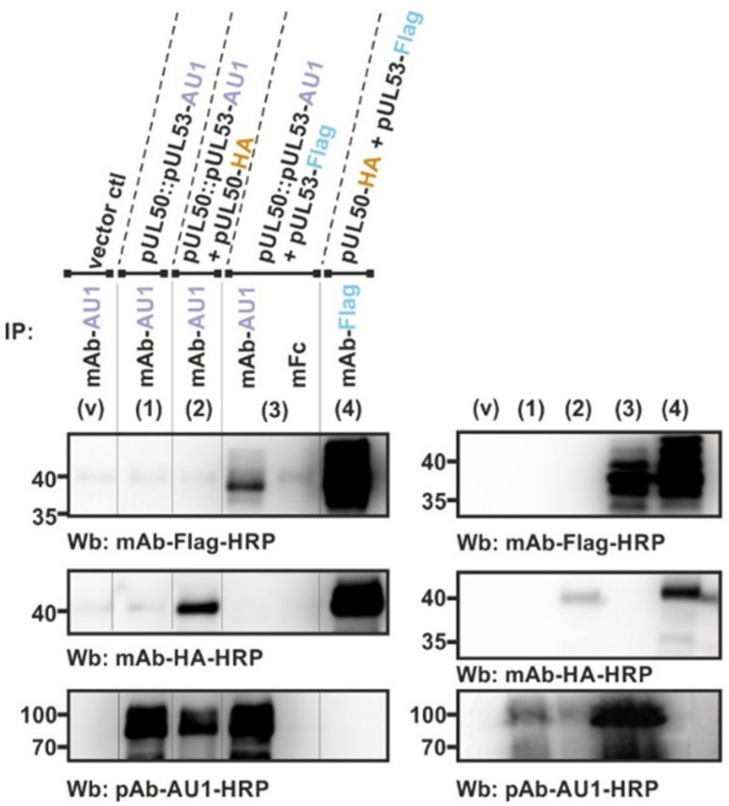
CoIP analysis demonstrating that a tagged fusion construct of the two HCMV core NEC proteins (pUL50::pUL53-AU1) is still capable of additional interactions. The indicated expression plasmids were used for the transient cotransfection of 293T cells and harvested at approx. 2 d p.t. for the preparation of total lysates. Expression levels of the individual proteins were monitored by the detection of lysate controls on Western blots (right panels: v, vector control; 1–4, coexpression protein samples, directly taken as the input for standard CoIP). The fusion protein alone (1) or combinations of twofold coexpression are indicated above the immunoblots (left panels: v, vector control; 2–4, coexpression lysates used for CoIP). Thereafter, the CoIP samples were washed and subsequently analyzed in a standard SDS-PAGE/Wb procedure, using the indicated antibodies for immunostaining (Wb). A nonreactive mouse Fc fragment (mFc, 3) was used as a negative control and the coexpression of pUL50-HA with pUL53-Flag (4) as a positive control.

The results obtained with the in vitro NEC assembly assay, suggesting hetero-oligomeric assembly properties, were confirmed by methodologically related assay conditions. For this purpose, a cotransfection experiment using the respective expression plasmids was performed to transiently coexpress the tagged versions of pUL50 and pUL53 at the indicated combinations in 293T cells. Total lysates were then produced for their direct use in a standard coimmunoprecipitation (CoIP) assay (Figure 3). The results of this coexpression setting were very similar to those of the in vitro assembly assay. Also, here, the fourfold (panel 2 and 7) or sixfold combinations (panel 3) were positive for interaction, and again threefold combinations with either three hook pUL53 versions alone (panel 4) or three groove pUL50 versions alone (panel 5) were negative. Likewise, the CoIP signals were mostly restricted to the Flag-specific IP (mAb-Flag), while HA-specific IP (mAb-HA) did produce much lower signal intensities of interaction. This was mostly assigned to a technical limitation (lower affinity of the mAb-HA or restricted accessibility of the HA tag), since a comparison of antibodies, using the threefold approach of coexpression, showed that mAb-AU1 was clearly positive in the detection of this complex (panels 6–7). In addition, the postulated binding pattern was further supported by the use of a pUL50::pUL53 fusion construct (Figure 4). This finding demonstrated that the fusion construct still folds into a conformation that is capable to further interact with NEC partners, putatively also forming hetero-oligomers. The coexpression of either pUL50-HA (Figure 4, lane 2) or pUL53-Flag (lane 3, left) provided a strongly positive CoIP signal. Thus, all data obtained with the two different systems of coexpression-based CoIP and in vitro assembly of the HCMV core NEC are consistent with the concept of oligomeric interaction.

### 3.4. Interface Analysis of the Hexameric NEC and Computational Investigation of pUL50 Conformational Stability

**Figure 5:**
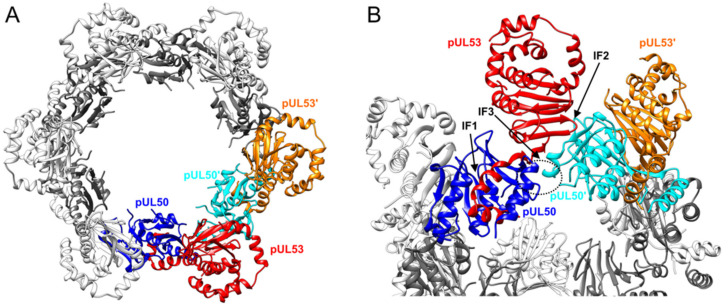
Crystal structure of the hexameric HCMV NEC. (**A**) Top view on the NEC highlighting two adjacent pUL50–pUL53 heterodimers in color. For the remaining building blocks, pUL50 and pUL53 are shown in grey and white, respectively. (**B**) Enlargement of the interface region. The most prominent interaction within the pUL50–pUL53 heterodimer is the hook-into-groove interface (labeled as IF1). Between adjacent pUL50–pUL53 heterodimers, the interfaces IF2 (pUL53–pUL50′) and IF3 (pUL50–pUL50′) are observed. The latter interface is highlighted by a dotted circle. The structure was obtained from PDB code 5D5N.

The experimental data derived from Figure 1, Figure 2, Figure 3 and Figure 4 strongly suggested that differentially tagged combinations of pUL50 and pUL53 are able to form higher-order oligomers, whereas neither pUL50 nor pUL53 alone did homo-oligomerize. To understand the molecular origin for this observation, we inspected the structure of the hexameric rings present in the crystal structure of the HCMV NEC (Figure 5A; [9]), which exhibits multiple interfaces between the individual subunits (Figure 5B). The pUL50–pUL53 heterodimer is mainly stabilized by the hook-into-groove interaction (IF1 in Figure 5B). Formation of the hexameric rings from these heterodimeric building blocks involves two major types of interaction, namely pUL53–pUL50′ (IF2 in Figure 5B) and pUL50–pUL50′ (IF3 in Figure 5B).

**Figure 6:**
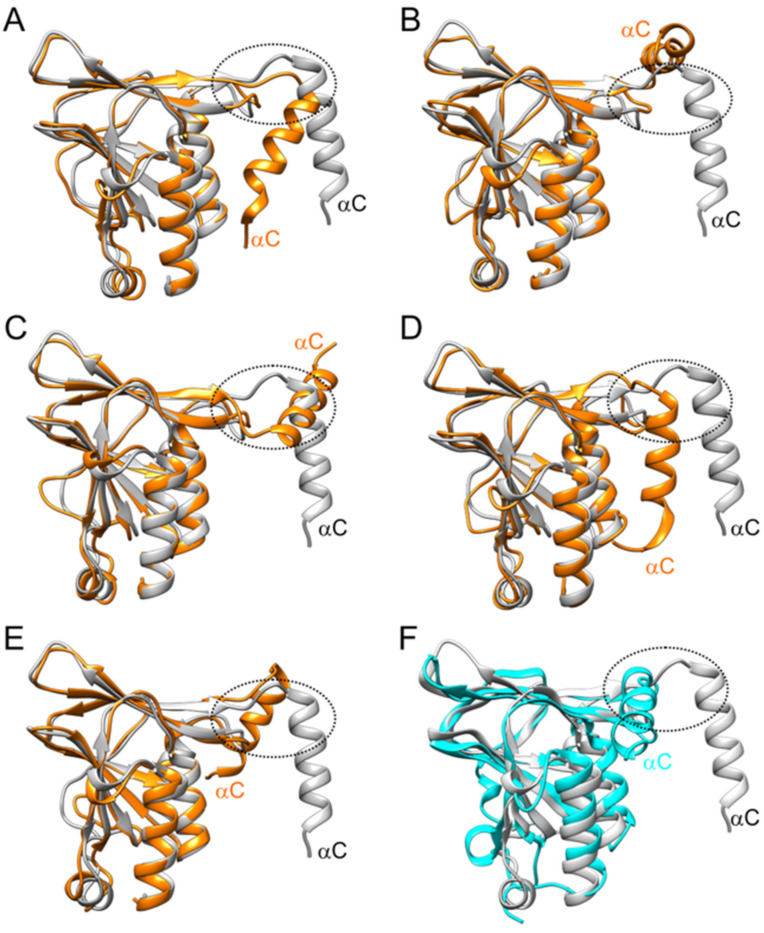
Conformational plasticity of pUL50 in the absence of pUL53. (**A**–**E**) Overlay of the pUL50 conformation present in the NEC (grey) with representative structures observed during the molecular dynamics simulations (orange) highlighting the conformational flexibility of helix αC. A dotted circle marks the region that is involved in the pUL50–pUL50′ interactions in the hexameric NEC (see Figure 5B for details). (**F**) Overlay of the pUL50 conformation in the NEC (grey) with the conformation observed for the unbound MCMV pM50 (cyan). The relevant structures were obtained from the PDB (codes 5D5N and 5A3G).

**Figure 7:**
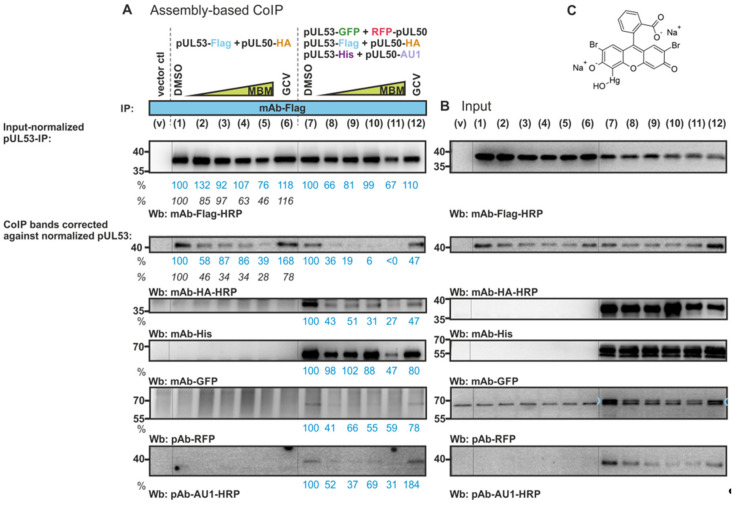
Analysis of the sensitivity of the HCMV core NEC oligomeric assembly towards a small molecule NEC inhibitor. (**A**) An in vitro NEC assembly assay was performed in the presence of increasing concentrations of the newly identified small molecule NEC inhibitor merbromin ([30]; MBM 1.25, 2.5, 5 and 10 µM; 0 µM/DMSO control). The indicated expression plasmids were used for the single transient transfection of 293T cells and harvested at approx. 2 d p.t. for the preparation of total lysates. Combinations of these samples were used in twofold or sixfold settings of in vitro assembly as indicated above the immunoblots (left panels: v, vector control; 1–12, combinations of in vitro assembly reactions; incubated at 4 °C overnight under rotation). Optionally, in vitro assembly reactions were performed in the presence of MBM (panels 2–5 and 8–11; 1.25–10 µM), DMSO as a solvent control (panels 1 and 7) or ganciclovir as an antiviral reference drug (GCV, 10 µM; panels 6 and 12). Thereafter, the assembled complexes were subjected to CoIP, using the indicated immunoprecipitation antibodies (IP), and subsequently analyzed in a standard SDS-PAGE/Wb procedure, using the indicated antibodies for immunostaining (Wb). Note in panels 1–6, the MBM-mediated reduction of pUL50-HA assembly (lowest Wb panel), which was not obtained with GCV. Note in panels 7–12, the MBM-mediated reduction of the assembly of His-, GFP-, RFP-, AU1- and HA-tagged versions of the NEC proteins (while pUL53-Flag, as the direct IP target of mAb-Flag, remained mostly unaffected). The GCV control showed either no or only modest reducing activity compared to DMSO. Quantitation of the band intensities was performed by densitometry (AIDA Image Analyzer v.4.23 software). All determinations were done in duplicate and the mean values of CoIP signals were at first corrected against the individual input levels (i.e., the DMSO controls were taken as reference bands and variations of protein input levels were calculated as correction factors that were then applied to adjust the corresponding values of CoIP bands). The second step of normalization was then performed through a correction factor that compensated the partly varying amounts of immunoprecipitated pUL53 (mAb-Flag-HRP) so that percentages referring to the DMSO control are given. For lanes 1–6, an independent replicate of this experiment (primary data not shown) was likewise evaluated as indicated by the additional values in italics. (**B**) Expression levels of the individual proteins were monitored by the detection of lysate controls on Western blots (right panels: v, vector control; 1–12, single-expression protein samples, subsequently taken as the input for in vitro assembly reactions). (**C**) Chemical structure of merbromin (MBM).

The absence of pUL50 homo-multimers in the experiments prompted us to investigate the conformational stability of isolated pUL50 in more detail. For this purpose, we performed molecular dynamics simulations starting from the pUL50 conformation observed in the hexameric ring (Figure 6). These simulations showed that the C-terminal helix αC, which is part of the pUL50–pUL50′ interface in the hexamer, was rather flexible and sampled multiple different conformations during the simulations (Figure 6A–E). Some of these conformations became stabilized by interactions between αC and the globular part of pUL50 (Figure 6D,E) and caused a distorted arrangement of αC compared to the pUL53-bound conformation. Such a distorted arrangement of αC has also been observed in the experimentally determined structure of the unbound MCMV pM50 (Figure 6F; [7]). Thus, pUL53 appears important for hexamer formation in two critical aspects: (i) to provide the hook-into-groove interaction (Figure 5B; IF1) that fixes pUL50-αC in a proper conformation for homomeric pUL50–pUL50′ interactions (Figure 5B; IF3), and (ii) to provide an additional platform for the formation of pUL53–pUL50′ heteromeric interactions (Figure 5B; IF2). Combined, this part of the study underscores the utmost importance of the pUL50–pUL53 interaction for the formation of a functional NEC, rendering this interaction an attractive drug target.

### 3.5. Blocking of In Vitro Assembly-Based Oligomeric pUL50-pUL53 Interaction by an NEC-Directed Inhibitor

Next, the question of the sensitivity of the HCMV core NEC assembly towards a small molecule NEC inhibitor was addressed (Figure 7A–C). For this purpose, another in vitro NEC assembly assay was performed in the presence of increasing concentrations of a newly identified NEC inhibitor termed merbromin (MBM, Figure 7C). MBM has recently been identified, using an in vitro binding inhibition assay (competitive ELISA) involving recombinant pUL50 and pUL53 [8], through the screening of the Prestwick Chemical Library^®^ (Illkirch-Graffenstaden, France; [30]). Here, analogously we performed NEC in vitro assembly in the presence of increasing concentrations of merbromin (1.25, 2.5, 5 and 10 µM of MBM; 0 µM/DMSO solvent control). In the control setting of this assay, Flag-specific staining (mAb-Flag-HRP) was used to monitor the MBM effect on pUL53 itself, which served as the IP target. Some modest level of pUL53-Flag reduction was noted (mostly at the highest concentration of 10 µM; Figure 7A, lane 11) and this was taken into account when normalizing the signals during the quantitative evaluation. It was unexpected to see some reduction of the IP target pUL53 in the presence of the drug, so this might indicate some secondary effect of MBM in the partial destabilization of drug-interacting proteins. In addition to this rather limited effect, in panels 1–6 or 7–12, a pronounced MBM-mediated reduction of NEC in vitro assembly was demonstrated for either twofold or sixfold protein combinations, respectively. In the twofold assembly setting (left part of Figure 7A), pUL50-HA interaction was reduced in dependence of the increasing MBM concentrations compared to DMSO (second uppermost Wb panel), while no reduction was obtained with GCV used as a drug selectivity control. In the sixfold assembly setting (right part of Figure 7A), the MBM-mediated reduction of the assembly of His-, GFP-, RFP-, AU1- and HA-tagged versions of the two NEC proteins was detected, while also here, the GCV control showed either no or only modest reducing activity. A quantitative evaluation, which was performed by densitometric analysis of the Western blot signals (mean values of MBM-mediated reduction given as percentages below the panels in blue letters), supported in part the described effect. Comparing the drug effects measured for the twofold and sixfold assemblies, it became evident that the twofold system produced clearer drug-mediated effects (see a second experimental replicate amended by additional densitometric values in italics; left part of Figure 7, panels 1–6), while the protein quantities of the sixfold system (panels 7–12) showed a higher degree of variation. Concerning the twofold system, a statistically significant MBM-mediated effect of reduced pUL50-HA versus the DMSO control was obtained for the drug concentrations of 1.25, 2.5 and 10 µM in the upper replicate (blue) and for all drug concentrations in the lower, second replicate (significance levels by Student’s *t*-test all at least *, *p* ≤ 0.05). Concerning the sixfold system, statistically significant MBM-mediated effects of reduced pUL50-HA and pUL53-His versus the DMSO control were obtained for all drug concentrations (significance levels of ***, *p* ≤ 0.001 or **, *p* ≤ 0.01), while the other tagged binding partners showed variation so that reduction was not consistently statistically significant (n.s., *p* > 0.05, for six of twelve values). Thus, it has to be emphasized that this measurement can only be regarded as a semi-quantitative assay and may rather provide a tendency than clear evidence. Nonetheless, these findings are compatible with MBM sensitivity and suggest for some of the measured binding properties an MBM effect on the oligomeric NEC assembly.

### 3.6. Interference of the Inhibitory Small Molecule MBM with NEC Nuclear Rim Formation and Viral Nuclear Egress Activity

**Figure 8:**
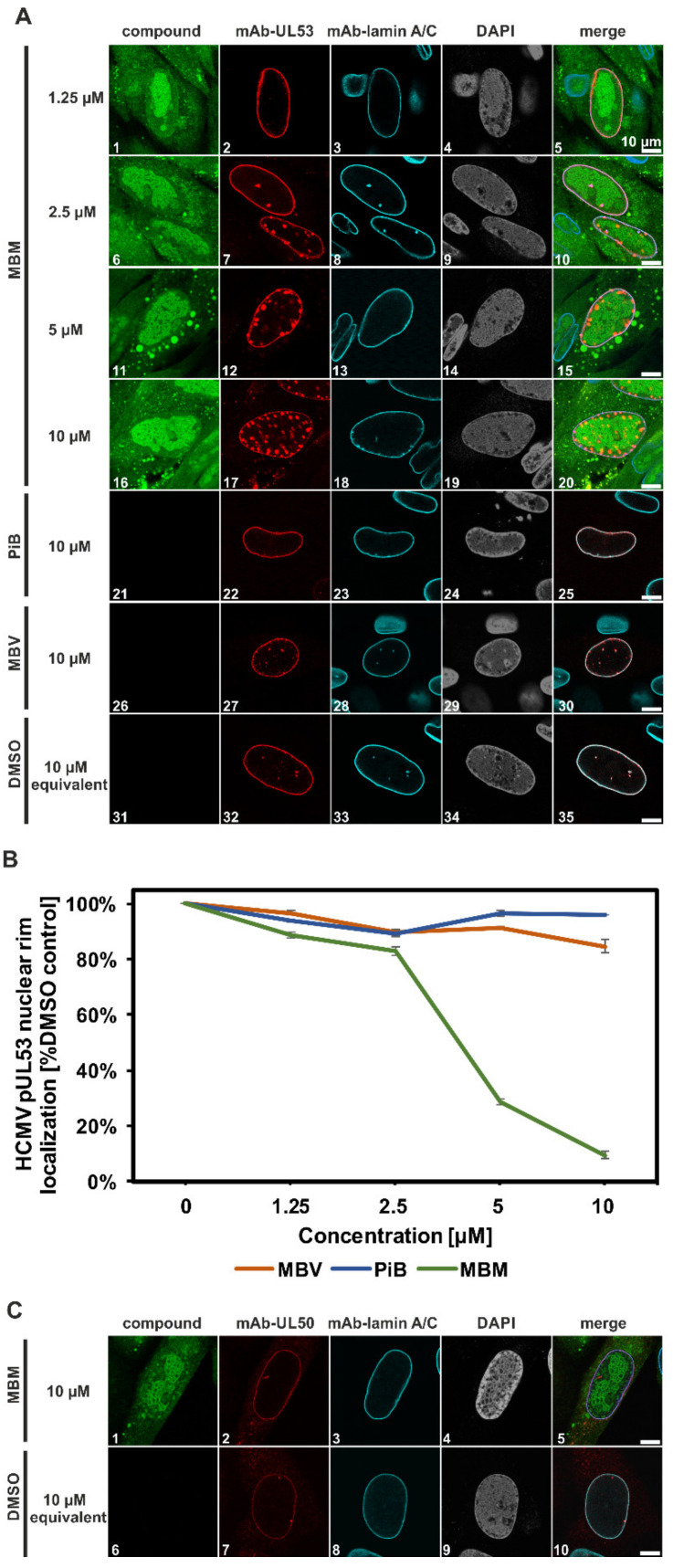
Confocal imaging of NEC nuclear rim formation in HCMV-infected fibroblasts under MBM treatment. (**A**) Human foreskin fibroblasts (HFFs) were cultivated in 6-well plates on cover slips, used for HCMV infection at a multiplicity of infection (MOI) of 0.1 and fixed at 5 d p.i. Indirect immunofluorescence staining was performed for viral pUL53 and cellular lamin A/C. Counterstaining of the autofluorescent drug (MBM) and the nuclei (DAPI) are indicated and a merge of all signals is given at the right. Note the concentration-dependent inhibition of normal pUL53 nuclear rim localization by MBM, but not by the control compounds PiB and maribavir (MBV). (**B**) Quantitation of the drug-mediated decrease of pUL53 nuclear rim localization was performed by visual microscopic counting. The criteria of counting were based on areas of pUL53-positive cells that either comprised a perfect nuclear rim localization of pUL53 (positive, i.e., identical with DMSO control) or a loss of perfect nuclear rim localization by the occurrence of intranuclear speckles (negative). Several areas of positive cells (68 cells in mean) were used for the evaluations and mean values ± SD of counting in triplicate are shown. (**C**) A comparative staining was performed for pUL50 under identical conditions. Note the lack of an MBM-mediated detachment of pUL50 from the nuclear rim.

The specificity of the MBM-mediated assembly blocking effect towards the viral NEC was assessed by additional confocal imaging experimentation (Figure 8). Here, the activity of MBM was compared to other NEC-relevant inhibitory drugs, i.e., PiB, an inhibitor of the cellular prolyl cis/trans isomerase Pin1 that possesses a nuclear egress-promoting function [37,38], and maribavir (MBV), an inhibitor of the major nuclear egress-regulating viral protein kinase pUL97 [39,40,41,42,43,44,45,46,47]. As an important result, a marked concentration-dependent effect was identified for MBM in terms of dislocating the nuclear rim association of pUL53, which was not seen for the other drugs. MBM treatment with a low concentration of 2.5 µM showed a slight effect of inducing the NEC dislocation of pUL53 in HCMV-infected cells (Figure 8A, panels 6–10; compare to DMSO control, panels 31–35), a dislocation effect that was found massively increased with higher concentrations of 5–10 µM. The 10 µM-MBM treatment induced an extensive detachment of pUL53 from the nuclear rim, resulting in a speckled relocalization of the main pUL53 fraction towards an internal nucleoplasmic localization (Figure 8A, panels 16–20). This effect could also be quantitatively assessed by microscopic counting, thereby confirming the concentration-dependent inhibition of pUL53 nuclear rim localization by MBM, which was statistically significant at 5 and 10 µM (Figure 8B). The PiB reference control was negative, while interestingly, MBV at high concentrations showed a slight effect on pUL53 localization. The latter point might reflect the pUL53-phosphorylating activity of pUL97 [45]. Since the viral kinase is targeted by MBV, this pUL97 inhibition may possibly correlate with a limited regulatory effect on pUL53 localization. To address the question whether pUL50, representing the inner nuclear membrane-bound component of the core NEC, may also be detached from the nuclear rim through MBM treatment, additional staining with mAb-UL50 was performed under identical conditions (Figure 8C). Neither an intermediate concentration of 2.5 µM (not shown) nor a high concentration of 10 µM of MBM (panels 1–10) induced a detachment of pUL50. Nuclear rim localization of pUL50 was found in the majority of infected cells under both MBM treatment (>99% of virus-positive nuclei) or DMSO control treatment (>99%). Among these cells, the visual counting of perfect pUL50 rim staining (27.9 ± 3.1% MBM 10 µM versus 35.8 ± 3.2% DMSO) and a pUL50 staining pattern that included the nuclear rim plus rim-independent signals (72.1 ± 3.1% MBM 10 µM versus 64.2 ± 3.2% DMSO) also did not reveal any pUL50-directed activity of MBM. In essence, these data demonstrate a strong activity exerted by MBM on the nuclear rim localization of pUL53, which is consistent with the blocking activity of MBM exerted on the in vitro core NEC assembly.

**Figure 9:**
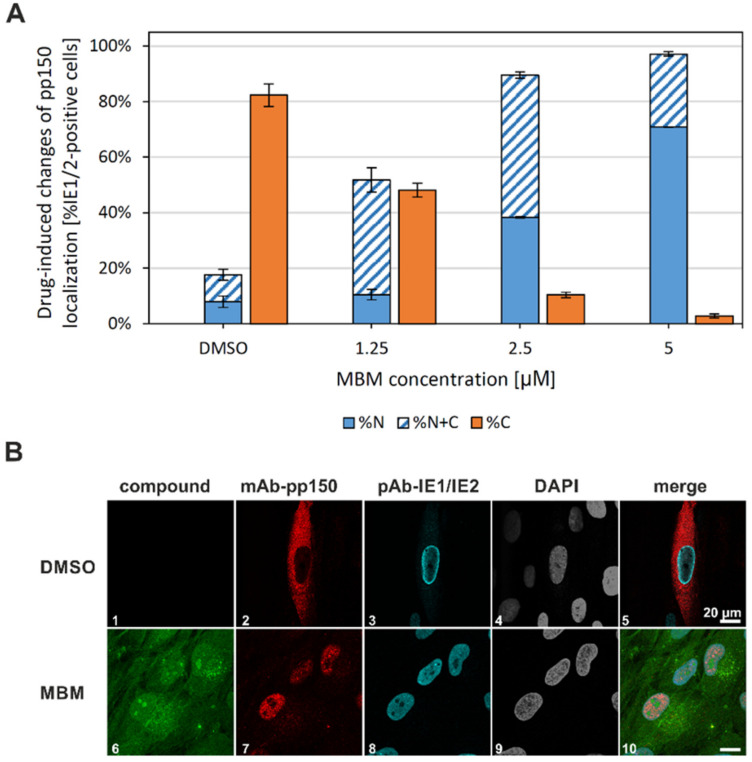
Confocal microscopic evaluation of cytoplasmic viral pp150 immunofluorescence signals in HCMV-infected cells in the absence or presence of MBM treatment (entire staining signals of pp150, independent of cytoplasmic virion assembly compartment (cVAC) structures). (**A**) HFFs were cultivated in 6-well plates on cover slips, used for HCMV infection at a MOI of 0.1 and fixed at 5 d p.i. Indirect immunofluorescence staining was performed for viral pp150 (pUL32). IE1/IE2 staining was used as an infected-cell control by far-red wavelength, as monitored in a separate microscopic channel. Nuclear counterstaining was additionally performed (DAPI) and the presence of the drug (MBM; 1.25–5 µM as indicated) was visualized by its autofluorescence. Several areas of positive cells (87 cells in mean) were used for the quantitation by visual microscopic counting; all counts were performed in duplicate and mean values ± standard error are given. N, nuclear localization of pp150; N+C nuclear-plus-cytoplasmic localization; C, purely cytoplasmic. (**B**) Characteristic staining patterns obtained for pp150, standing as exemplar signals used for the microscopic evaluation. MBM, merbromin 5 µM; DMSO, solvent control without MBM; scale bar marks 20 µm.

Another point strongly supporting the NEC-directed inhibitory activity of MBM is its antiviral activity measured for HCMV replication in HFFs by using various readout systems. The average antiviral EC_50_ value, determined as a mean of three different experimental settings, was calculated as 3.2 ± 0.6 µM [30]. In order to analyze this antiviral activity of MBM in more mechanistic terms, in particular, to correlate antiviral activity with the in vitro inhibition of NEC assembly, a confocal imaging-based evaluation of the HCMV nuclear egress activity was performed as methodologically described before [5]. To this end, an indirect immunofluorescence staining of the viral tegument protein pp150 (pUL32) was performed in HCMV-infected HFFs under treatment with increasing concentrations of MBM. HCMV pp150 was considered as an attractive marker protein, as it has been found closely associated with viral capsids when determining the atomic structure [48], and its incorporation into nascent HCMV particles occurred already in the infected-cell nuclei during nuclear assembly [49]. It could thus be used as a marker for the tracking of viral nucleocytoplasmic capsid egress [44], after which the pp150 signals typically accumulate in cytoplasmic virion assembly compartments (cVACs) [5,50]. By the use of mAb-pp150, we were able to detect a marked drug-induced change of the intracellular distribution of pp150 signals (Figure 9). In this case, the entire staining signals of pp150 were used for evaluation, independent of whether pp150 was found in a smooth cytoplasmic or nuclear localization or was contained in cVAC structures. Notably, the fraction of purely cytoplasmic pp150 signals in virus-infected cells (%C) decreased in an MBM concentration-dependent manner (Figure 9A; note that IE1/IE2-positive counterstaining was used to monitor the total of infected cells). The microscopic counting included all infected cells, and in the untreated situation (DMSO), the majority of cells comprised exclusively cytoplasmic pp150 staining. Although the distinction between purely nuclear (%N) and nuclear-plus-cytoplasmic (%N + C) cell populations was difficult; also, the sum of these two fractions pointed to a drug-mediated effect of MBM, resulting in nuclear retention of pp150. Exemplary staining panels illustrate the basis of the microscopic evaluation (Figure 9B). Note the cytoplasmic distribution of pp150 in infected cells without the drug (panels 1–5; DMSO), as compared to the nuclear retention upon MBM treatment (panels 6–10; MBM 5 µM). This finding suggests a first, indirect indication that the NEC-directed inhibitory potential of MBM may translate into a blocking activity of nuclear capsid egress. Further experimentation will be required to substantiate this conclusion.

**Figure 10:**
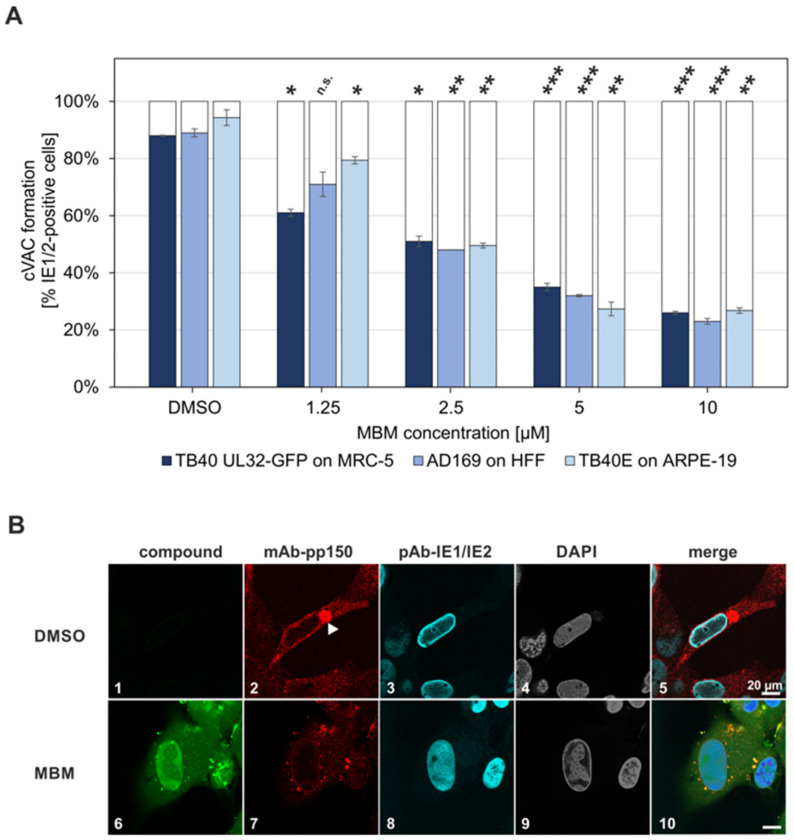
Confocal microscopic evaluation of pp150-positive cVAC formation in HCMV-infected cells in the absence or presence of MBM treatment. (**A**) Three different human cell types, namely MRC-5, HFF and ARPE-19, were cultivated in 6-well plates on cover slips and used for the infection with three different strains of HCMV (MOI ≤ 0.1) and fixed at 5 d p.i. Indirect immunofluorescence staining was performed for viral pp150 (pUL32), representing a marker of viral cVAC formation, and IE1/IE2 proteins, used as an infected-cell control. Nuclear counterstaining was additionally performed (DAPI) and the presence of the drug (MBM; 1.25–10 µM as indicated) was visualized by its autofluorescence. The formation of pp150-positive cVACs was applied for visual microscopic counting. The criteria of counting were based on the distinction between cells carrying fully-shaped cVAC structure and those lacking cVACs but showing dispersed pp150 signals. Several areas of positive cells were used for the quantitation by visual microscopic counting (134 cells in mean for TB40 UL32-GFP, 97 cells for AD169, 99 cells for TB40E); all counts were performed in duplicate and mean values ± standard error are given. Student’s t-test was applied to determine statistical significance (***, *p* ≤ 0.001; **, *p* ≤ 0.01; *, *p* ≤ 0.05; n.s., *p* > 0.05). (**B**) Exemplary images showing a fully-shaped cVAC structure in HFFs infected with HCMV AD169 (DMSO) and the lack of cVAC formation under MBM treatment (10 µM; for the entire set of raw data, see Figure S5). MBM, merbromin; DMSO, solvent control without MBM; scale bar marks 20 µm.

In the subsequent setting, specific focus was directed to the formation of cytoplasmic cVAC structures, also detected by immunofluorescence staining of pp150 representing one of the cVAC marker proteins (Figure 10). Hereby, the question was addressed whether drug treatment may limit the cytoplasmic appearance of pp150-positive viral capsids contained within cVACs, as a result of reduced nucleocytoplasmic egress. The methodological basis of cVAC quantitation has been described in our earlier report [5]. Using this approach, untreated cells (DMSO) comprised typical, fully-shaped cVAC structures in a large percentage of the infected cells, whereas MBM treatment reduced cVAC formation. In a concentration-dependent manner, MBM led to a reduction of cells carrying cVACs and to an increase of the cell fraction that lacked cVACs but exclusively showed dispersed pp150 signals (Figure 10A,B). This effect could be reproduced for three different viral strains (i.e., HCMV AD169, TB40 and Merlin) used for infection of three HCMV-permissive cell types (i.e., fibroblasts of the types HFF and MRC-5 and ARPE-19 epithelial cells). It should be noted that the setting with HCMV Merlin infection of ARPE-19 was restricted to a low number of positive cells, due to a low infectious titer of the Merlin stock virus so that quantitative assessment was not possible and an MBM-mediated reduction of cVACs could only be visualized for individual cells (data not shown). The signal for the cVAC marker pp150 was either obtained through indirect immunofluorescence staining (Figure 10B, panels 1–10) or autofluorescence of the pp150-GFP expressed by the virus recombinant TB40 UL32-GFP (Figure S5, panels 1–25) [44,49]. Importantly, in all cases, MBM treatment produced a marked reduction of cytoplasmic cVAC formation (Figure 10A; the entire set of raw data is shown in Figure S5). The inhibitory effect was statistically significant for the MBM concentrations of 2.5–10 µM in all three settings (significance levels of ***, *p* ≤ 0.001 or **, *p* ≤ 0.01). Combined, the findings are compatible with our statement that MBM inhibits the process of viral nuclear egress.

Finally, the question of a putative MBM effect on the formation of the multicomponent NEC, including cellular proteins associated with the viral core NEC, was addressed. In this context, it should be noted that cellular NEC-associated factors identified in the previous studies of our group and other researchers ([4] and references therein) were considered to undergo upregulation upon HCMV infection. This was exemplified by a Wb-/densitometry-based semi-quantitative analysis of the proteins p32/gC1qR, emerin, CDK1 and PKCα (Figures S1 and S2) in HCMV-infected primary human fibroblasts (HFFs). By using three different multiplicities of infection (MOI) with two different strains of HCMV (AD169 and TB40), a strong tendency of upregulation was found for these host factors in the period between 2–5 d p.i. Although some variation became evident in this measurement (e.g., for PKCα on d 5, Figure S1A, or emerin on d 2, Figure S1B), this increase in protein levels was mostly seen for the MOI of 0.1 and 0.5, whereas for a MOI of 2, the levels decreased in many cases, which may be due to the virus-induced cell lysis. The latter point is supported by the primary Wb staining patterns shown in Figure S2, when comparing cell- or virus-specific bands between a MOI of 2 and lower MOIs.

As far as the nuclear rim colocalization of these cellular proteins with the viral core NEC pUL50-pUL53 was concerned, firstly, a coexpression and costaining analysis was performed by confocal imaging using plasmid-transfected HeLa cells (Figure S3A). Cellular protein expression patterns were investigated in cells transfected with empty vector (Figure S3B), constructs for coexpression of pUL50 and pUL53 (Figure S3C), constructs for single expression of pUL50 or pUL53 (Figure S3D), or pUL50::pUL53 fusion constructs (Figure S3E). Pronounced colocalization signals between the nuclear rim staining of viral NEC proteins and the cellular factors were obtained for emerin (Figure S3C, panels 1–5), p32/gC1qR (panels 6–10 and in part for PKCα (panels 16–20), whereas CDK1 showed very little or no signals of NEC colocalization (panels 11–15). This colocalization pattern was similarly found using either coexpressed pUL50–pUL53 (Figure S3C), a single expression of pUL50 or pUL53 (Figure S3D) or the pUL50::pUL53 fusion construct (Figure S3E). It should be mentioned that for most of the cellular proteins analyzed (with the exception of the perfectly colocalized emerin), the degree of NEC colocalization was restricted to distinct staining areas (see enlarged insets shown in Figure S3E, panels 4, 9, 14, 19 and 24) or remained invisible in case of CDK1, although its capability to undergo NEC association has been demonstrated before [25,26]. In particular, this aspect is interesting for the example of p32/gC1qR, which is a mostly mitochondria-localized protein [51,52], but a fraction of which can also enter the nucleus as regulated through protein phosphorylation [53,54]. Recently, we reported an association of p32/gC1qR with the HCMV-specific NEC as detected by mass spectrometry-based proteomics and several other techniques [5,18,25,55]. The resolution of the crystal structure of p32/gC1qR and its biochemical characterization illustrated the pronounced capacity of dimerization, oligomerization and the interaction with a number of viral proteins, so that its role as a bridging factor in the multicomponent NEC has thus been postulated and substantiated by data of independent reports [4,55,56,57,58,59,60,61]. Next, also HCMV-infected HFFs were used for a localization analysis of these cellular proteins, and in a second step, for addressing putative MBM effects on changes of localization (Figure S4). Viral immediate early protein was used as a marker to track infected cells (Figure S4, pAb-IE1/IE2). The four NEC-associated cellular proteins showed almost identical localization patterns in HCMV-infected HFFs as in plasmid-transfected HeLa cells (compare Figures S3 and S4) and a perfect nuclear envelope localization was marked by emerin (Figure S4, panels 2, 7 and 12). Interestingly, concerning MBM treatment, the number of virus-positive cells was reduced, but no MBM-specific change in the localization of any of the four cellular factors could be detected as compared to the DMSO solvent controls of HCMV-infected or mock-infected cells (Figure S4, emerin in panels 1–15, p32/gC1qR in panels 16–30, CDK1 in panels 31–45, PKCα in panels 46–60; note that likewise, the MBM treatment of mock-infected cells did not change these localization patterns, data not shown). It should be emphasized, however, that these cellular factors are all multifunctional, a fact also expressed by their multiple intracellular localization sites (nuclear, cytoplasmic, membrane-associated or local accumulations). Therefore, only a mere fraction of their total protein levels may be associated with the viral NEC. Consequently, a drug-induced impairment of these proteins’ NEC-relevant functionality may not be visible through a change of intracellular localization. Importantly, however, this picture was clearly different from that obtained for the dislocation effect of MBM onto the viral NEC protein pUL53 itself, as shown above. Combined, our data support the statement that the HCMV-specific core NEC is a rate-limiting regulator of viral replication, including the newly detected properties of the oligomeric assembly. Thus, the NEC represents a validated drug target that shows sensitivity to small inhibitory molecules.

## 4. Discussion

With the present study, we provide a closer insight into the interaction properties of the cytomegalovirus core NEC. Using the tools provided by a panel of tagged NEC proteins, an in vitro NEC assembly assay was established. Moreover, by the application of a newly identified NEC-directed small molecule, we demonstrate the sensitivity of NEC assembly and the intracellular core NEC formation towards this inhibitor. Specifically, our data strongly suggest the following scenario: The HCMV pUL50–pUL53 heterodimer, which builds hexameric lattices for capsid binding in infected cells, shows a pronounced tendency to oligomerize in vitro (assembly assay) and in transiently cotransfected cells (conventional tag-specific CoIP). This finding was altogether surprising, since indeed we already knew that the tagging of HCMV core NEC proteins did not interfere with pUL50–pUL53 heterodimerization (neither in transient interaction settings nor in infection experiments with recombinant viruses [18,62]), but it could not be presupposed that tags would not interfere with oligomeric assemblies. Moreover, the use of a pUL50::pUL53 fusion construct, similar to the bacterially produced construct recently employed to determine a high-resolution hook-into-groove structure [8], also exhibited the tendency to form higher-order oligomers, indicating that a fusion of both proteins does not negatively influence these interaction properties. Cellular NEC-associated proteins were additionally recruited to the tagged core NECs. Furthermore, a small inhibitory molecule, which blocks the pUL50–pUL53 interaction in vitro, was likewise active in blocking NEC assembly in vitro as well as viral replication in HCMV-infected fibroblasts.

Interestingly, the oligomeric NEC interaction property could only be demonstrated in those cases of the in vitro assembly conditions, when both hook and groove components of the tagged NEC proteins were present. In other cases, in which either exclusively hook pUL53 protein versions or exclusively groove pUL50 protein versions were subjected to the assembly reaction, the result was negative. This appeared surprising, at least as far as the groove components are concerned, since the 3D structure of the HCMV core NEC revealed a ring-like arrangement of the pUL50 subunits. The hexameric assemblies are mainly stabilized by homomeric pUL50–pUL50′ and by heteromeric pUL53–pUL50′ interactions between adjacent subunits (Figure 5). The lack of significant pUL53–pUL53′ interactions suggests that this protein may not form larger homo-oligomers. This finding appears particularly interesting against the scientific background that a strong tendency of in vitro homodimerization of bacterially produced pUL53 has been observed by our group and other researchers before [9,63]. According to the experimental data presented in this study and earlier data derived from yeast two-hybrid analyses [32], no indication of homodimerization was obtained when pUL53 was expressed in eukaryotic cells. Specifically, Figure 2 and Figure 3 suggested that transient transfection-derived pUL53 does not homo-oligomerize. The situation might be different for pUL50, which appears to exhibit homomeric interactions in the NEC hexameric structure. However, the experimental data shown here demonstrate that also pUL50 alone, similar to pUL53, lacks the potential to form higher-order oligomers. We speculated that this observation might be due to the conformational instability of pUL50 in the absence of pUL53. This idea is supported by molecular dynamics simulations indicating that the position of the C-terminal helix αC in isolated pUL50 differs significantly from the conformation observed in the hexameric NEC (Figure 6). Thus, the finding suggests that the proper pUL50 conformation for hexamer formation is only adopted after interaction with pUL53. In addition, pUL53 is likely to play a second role in hexamer formation by strengthening the pUL50–pUL50′ interface by additional pUL53–pUL50′ interactions (Figure 5B).

As a future perspective of the present findings, the question should be addressed whether the NEC hexamer formation is a specific property of individual herpesviruses or a general feature shared by all herpesviruses. The answer to this question might not only specify the molecular mode of the structure–function relationship of herpesviral NECs but may also have implications on further antiviral research with NEC-directed inhibitors. Given the possibility that the contact interfaces of the core NEC hook and groove constituents may be highly similar between herpesviruses, in terms of both heterodimeric and oligomeric interactions, an inhibitor blocking such interaction might comprise a quite broad targeting potential. Thus, in the first-mentioned case of NEC-binding differences between individual herpesviruses, such inhibitor development may progress towards candidates of selective NEC-directed antivirals, whereas the latter case may support the idea of developing anti-herpesviral drugs with broad-spectrum activity. Although this approach of the generation of a novel type of NEC-directed antivirals still appears in its infancy, it has not escaped our notice that this approach may combine several attractive drug qualities. These may include a novel mechanism of blocking protein–protein interactions, a putative broad-spectrum activity, the targeting of a key-point of virus–host interaction and intracellular trapping of immunologically active antigens. Thus, further studies, based on an interdisciplinary platform focusing the NEC-specific binding properties, functions and inhibitor sensitivity will be required to extend this current state of knowledge.

## Data Availability

The responsible authors declare that this article fully complies with the Data Availability Statements in section “MDPI Research Data Policies” at https://www.mdpi.com/ethics (accessed on 12 March 2021).

## Supplementary Materials

The following are available online at https://www.mdpi.com/1999-4915/13/3/462/s1. Table S1, Oligonucleotide primers used in this study; Figure S1, Regulatory effect of HCMV infection on the expression levels of NEC-associated cellular factors in dependence of viral strains and the MOI of infection: quantitation by densitometry of Western blot detection; Figure S2, Regulatory effect of HCMV infection on the expression levels of NEC-associated cellular factors in dependence of viral strains and the MOI of infection; Figure S3, Expression of tagged versions of HCMV core NEC proteins in regard of the nuclear localization of NEC-associated cellular factors; Figure S4, Lack of an effect of MBM on the nuclear localization of NEC-associated cellular factors; Figure S5, Confocal microscopic evaluation of pp150-positive cVAC formation in HCMV-infected cells in the absence and presence of MBM treatment.
