# Supplementary material for: Properties of Oligomeric Interaction of the Cytomegalovirus Core Nuclear Egress Complex (NEC) and Its Sensitivity to an NEC Inhibitory Small Molecule
Source: Viruses. 2021 Mar 11;13(3):462. doi: 10.3390/v13030462 (PMC8002134; doi:10.3390/v13030462)
Supplement: Supplementary file 1 [file viruses-13-00462-s001.pdf]

viruses

MDPI

Supplementary Materials

Properties of oligomeric interaction of the cytomegalovirus core nuclear egress complex (NEC) and its sensitivity to an NEC inhibitory small molecule

Jintawee Kicuntod[1], Sewar Alkhashrom[2], Sigrun Häge[1], Benedikt Diewald[3], Regina Müller[1], Friedrich Hahn[1], Peter Lischka[4], Heinrich Sticht[3], Jutta Eichler[2] & Manfred Marschall[1,*]

[1] Institute for Clinical and Molecular Virology, Friedrich-Alexander University of Erlangen-Nürnberg (FAU), Erlangen, Germany; jintawee.kicuntod@extern.uk-erlangen.de (J.K.), sigrun.haege@fau.de (S.H.), friedrich.hahn@uk-erlangen.de (F.H.), mueller.regina@uk-erlangen.de (R.M.), manfred.marschall@fau.de (M.M.)

[2] Department of Chemistry and Pharmacy, Division of Medicinal Chemistry, FAU Erlangen-Nürnberg, Erlangen, Germany; sewar.alkhashrom@fau.de (S.A.), jutta.eichler@fau.de (J.E.)

[3] Division of Bioinformatics, Institute of Biochemistry, FAU, Erlangen-Nürnberg, Germany; benedikt@diewald.com (B.D.), heinrich.sticht@fau.de (H.S.)

[4] AiCuris Anti-Infective Cures GmbH, Wuppertal, Germany; peter.lischka@aicuris.com (P.L.)

* Correspondence: e-mail manfred.marschall@fau.de, phone +49 9131 8526089

Table S1. Oligonucleotide primers used in this study.

Constructs	Primers	No. of nts.	Sequences
pUL50-HA	5'-UL50-BamHI-EcoRI	42	TGAGGATCCAGGAATTCATGGAGATGAACAAGGTTCTCCATC
	3'-UL50-*HA*-XhoI-PstI	67	TGACTGCAGCTCGAGTCA*AGCGTAATCTGGAACATCGTATGGGTA*GTCGCGGTGTGCGGAGCGTGTC
pUL50-RFP	5'-UL50-EcoRI	34	TGAGAATTCATGGAGATGAACAAGGTTCTCCATC
	3'-UL50-BamHI	35	TGAGGATCCCGGTCGCGGTGTGCGGAGCGTGTCGG
pUL50-AU1	5'-UL50-EcoRI	34	TGAGAATTCATGGAGATGAACAAGGTTCTCCATC
	3'-UL50-*AU1*-XhoI	46	GAACCTTGTTCATCTC*GATGTAGCGGTAGGTGTC*CATGAATTCCTA
pUL53-GFP	5'-UL53-EcoRI	31	TGAGAATTCATGTCTAGCGTGAGCGGCGTGC
	3'-UL53-BamHI	32	TGAGGATCCCGAGGCGCACGAATGCTGTTGAG
pUL53-Flag	5'-UL53-BamHI-EcoRI	39	TGAGGATCCAGGAATTCATGTCTAGCGTGAGCGGCGTGC
	3'-UL53-*FLAG*-XhoI-PstI	70	TGACTGCAGCTCGAGTCA*CTTGTCGTCATCGTCTTTGTAGTC*AGGCGCACGAATGCTGTTGAGAAACAGC
pUL53-His	5'-UL53-EcoRI	31	TGAGAATTCATGTCTAGCGTGAGCGGCGTGC
	3'-UL53-*His*-XhoI	49	TAGCTCGAGTCA*ATGGTGATGGTGATGATG*AGGCGCACGAATGCTGTTG
pUL50::pUL53-AU1	5'-UL50-EcoRI	34	TGAGAATTCATGGAGATGAACAAGGTTCTCCATC
	3'-UL50(linker)-BamHI	23	TAGGGATCCCCCGTCGCGGTGTG
	5'-UL53(linker)-BamHI	29	TAGGGATCCGGGAGCATGTCTAGCGTGAG
	3'-UL53-*AU1*-XhoI	51	TAGCTCGAGTCA*GATGTAGCGGTAGGTGTC*AGGCGCACGAATGCTGTTGAG

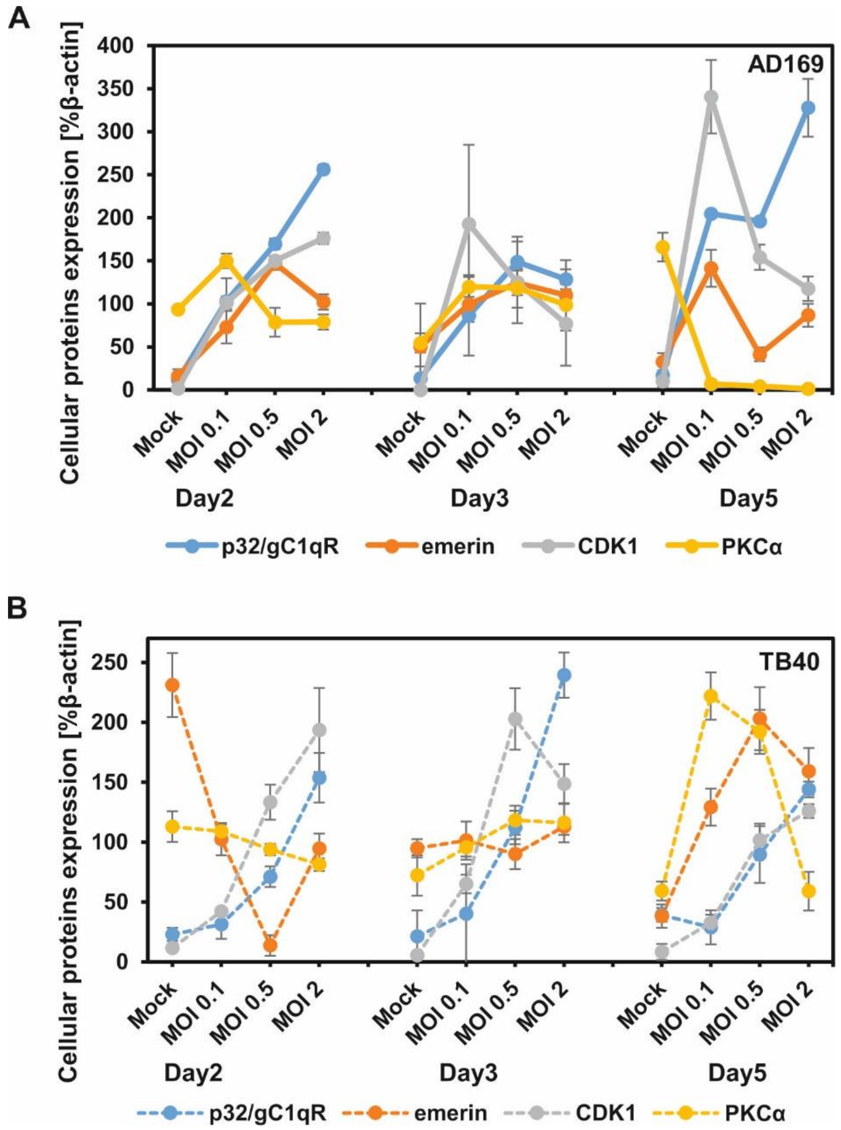

Figure S1. Regulatory effect of HCMV infection on the expression levels of NEC-associated cellular factors in dependence of viral strains and the MOI of infection: quantitation by densitometry of Western blot detection. HFFs were cultivated in 12-well plates and used for infection with two strains of HCMV, AD169 **(A)** and TB40 **(B)**, at the multiplicity of infection (MOI) indicated. At the time points 2, 3 and 5 days post-infection (d p.i.), cells were harvested and used for SDS-PAGE/Wb analysis. The antibodies used for the detection of cellular proteins were as described in Materials and Methods section. Each experiment was performed in three replicates and one representative replicate was used for the determination of protein expression levels by densitometry using Wbs as exemplary shown in Fig. S2. Each densitometric determination was performed in quadruplicate (two Wbs from identical samples, densitometry in duplicate).

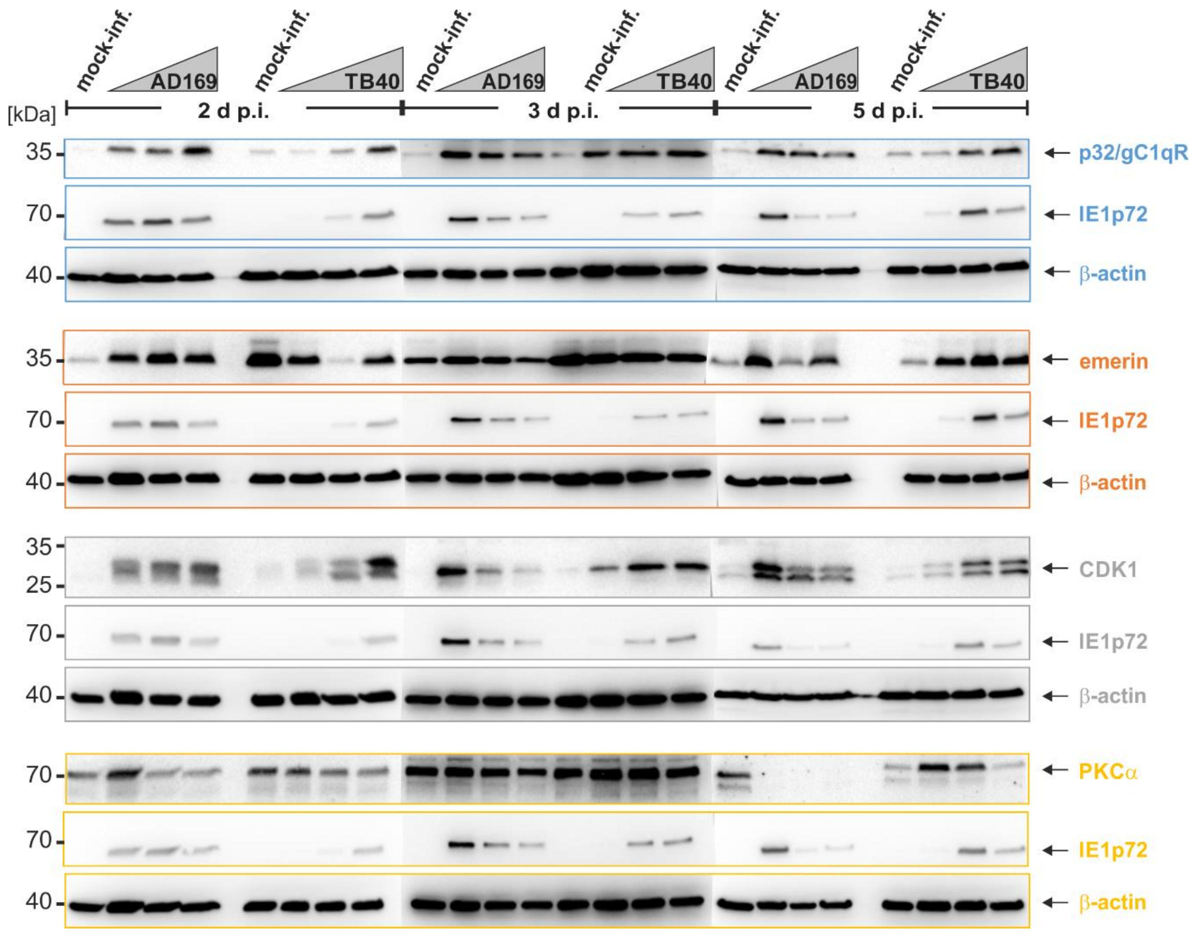

Figure S2. Regulatory effect of HCMV infection on the expression levels of NEC-associated cellular factors in dependence of viral strains and the MOI of infection. HFFs were cultivated in 12-well plates and used for infection with two strains of HCMV, AD169 and TB40, at the MOIs of 0.1, 0.5 and 2. At the time points 2, 3 and 5 d p.i., cells were harvested and used for SDS-PAGE/Wb analysis. The antibodies used for the detection of cellular proteins were as described in Materials and Methods section. As a viral marker protein, IE1p72 was counterstained for each set of panels. Comparative illustration of Western blot stainings: orange, p32/gC1qR; blue, emerin; violet, CDK1; green, PKCα.

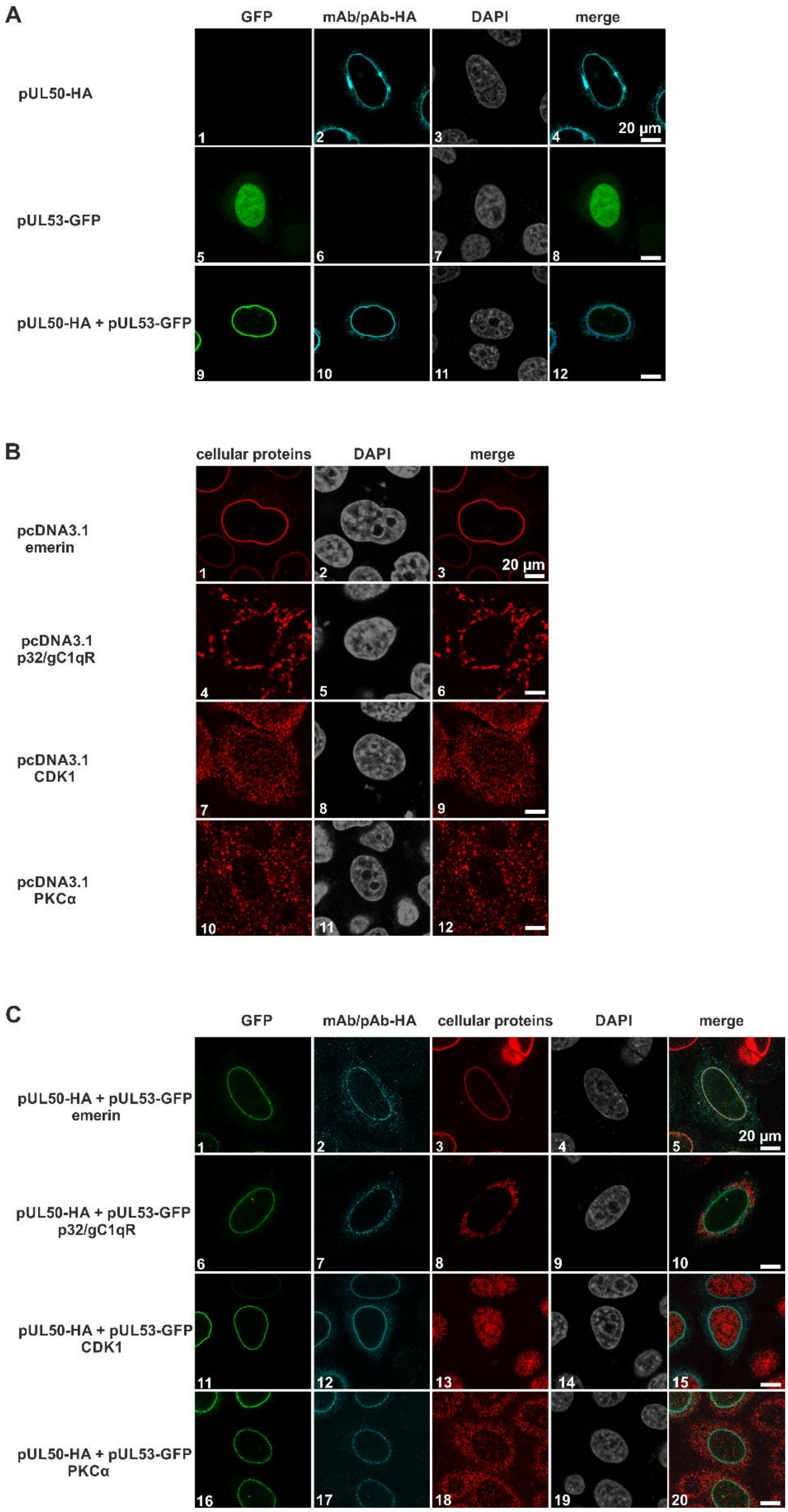
A
GFP
mAb/pAb-HA
DAPI
merge
pUL50-HA
pUL53-GFP
pUL50-HA + pUL53-GFP
20 µm
B
cellular proteins
DAPI
merge
pcDNA3.1 emerin
pcDNA3.1 p32/gC1qR
pcDNA3.1 CDK1
pcDNA3.1 PKCα
20 µm
C
GFP
mAb/pAb-HA
cellular proteins
DAPI
merge
pUL50-HA + pUL53-GFP emerin
pUL50-HA + pUL53-GFP p32/gC1qR
pUL50-HA + pUL53-GFP CDK1
pUL50-HA + pUL53-GFP PKCα
20 µm

D

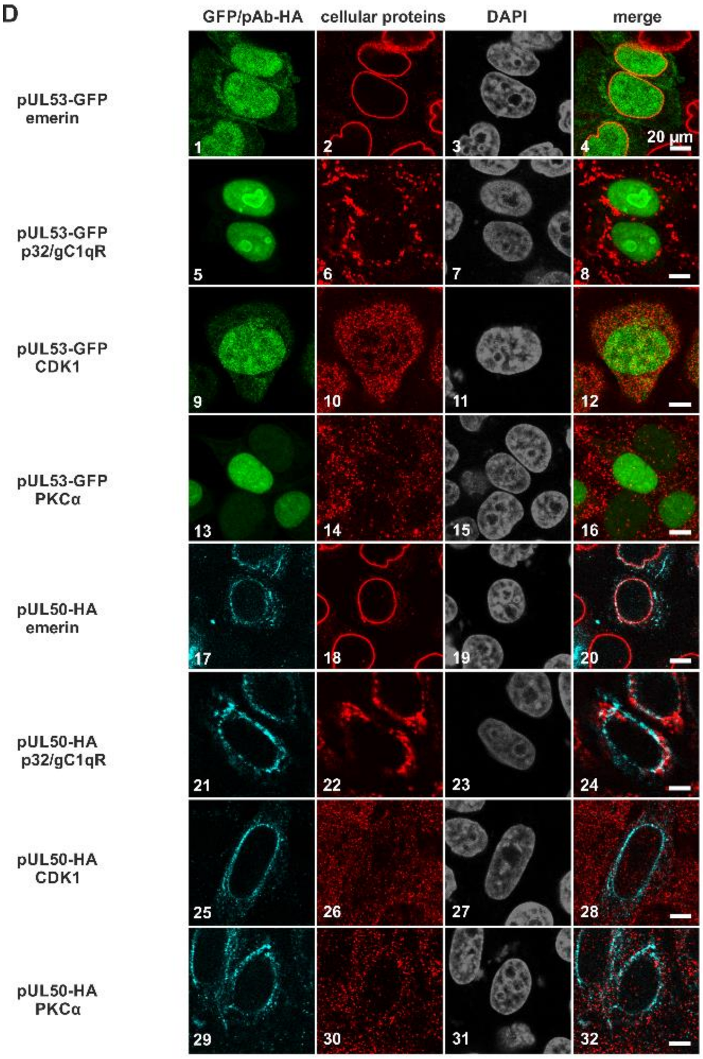
GFP/pAb-HA
cellular proteins
DAPI
merge
pUL53-GFP emerin
pUL53-GFP p32/gC1qR
pUL53-GFP CDK1
pUL53-GFP PKCα
pUL50-HA emerin
pUL50-HA p32/gC1qR
pUL50-HA CDK1
pUL50-HA PKCα
20 µm

E

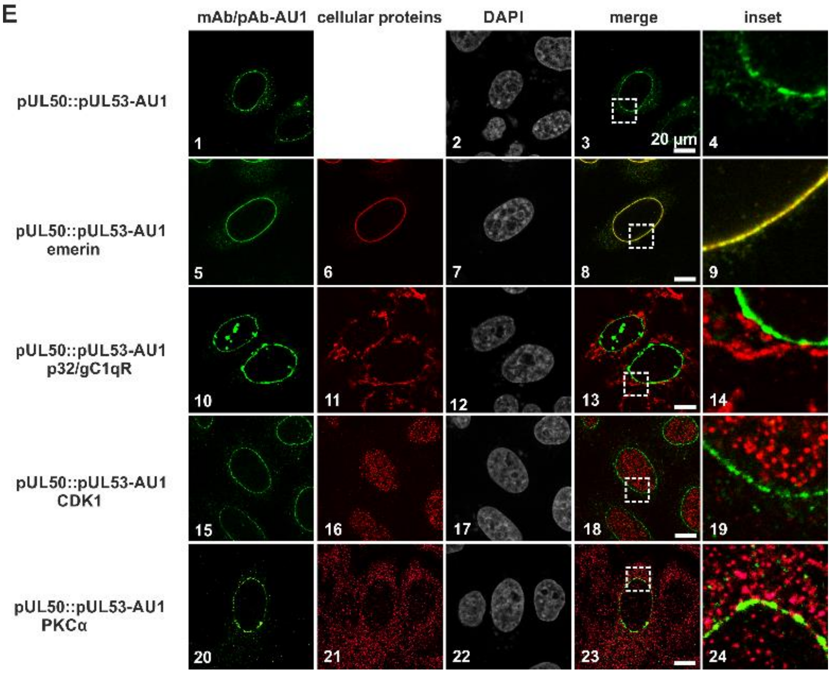
mAb/pAb-AU1
cellular proteins
DAPI
merge
inset
pUL50::pUL53-AU1
pUL50::pUL53-AU1 emerin
pUL50::pUL53-AU1 p32/gC1qR
pUL50::pUL53-AU1 CDK1
pUL50::pUL53-AU1 PKCα
20 µm

Figure S3. Expression of tagged versions of HCMV core NEC proteins in regard of the nuclear localization of NEC-associated cellular factors. Confocal imaging analysis was performed by the use of HeLa cells transiently transfected in 6-well plates with the indicated expression constructs for pUL50 and pUL53. After 2 days, cells were fixed and used for indirect immunofluorescence staining of cellular proteins: emerin, p32/gC1qR, CDK1 and PKCα. DAPI was used for nuclear counterstaining. Note, the colocalization between cellular proteins and viral NEC proteins in specific cases. Insets were used to highlight example of colocalization. **(A)** Single or cotransfection with constructs for pUL50 and pUL53; **(B)** transfection controls with empty vector (pcDNA3.1); **(C)** localization patterns of four cellular proteins upon pUL50 and pUL53 cotransfection; **(D)** localization patterns of four cellular proteins upon single transfection of pUL50 or pUL53; **(E)** transfection of the fusion construct pUL50::pUL53-AU1.

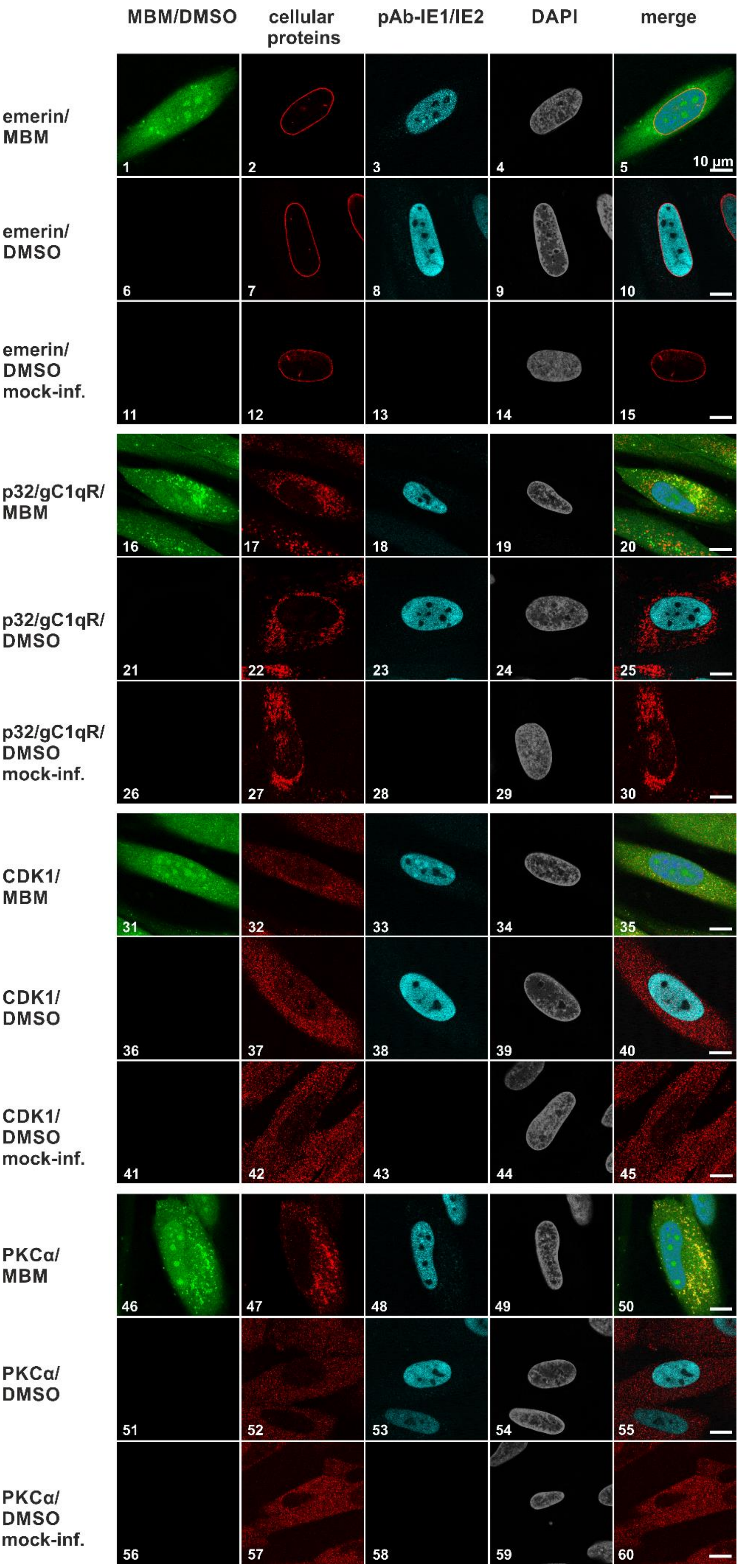
MBM/DMSO
cellular proteins
pAb-IE1/IE2
DAPI
merge
emerin/ MBM
emerin/ DMSO
emerin/ DMSO mock-inf.
p32/gC1qR/ MBM
p32/gC1qR/ DMSO
p32/gC1qR/ DMSO mock-inf.
CDK1/ MBM
CDK1/ DMSO
CDK1/ DMSO mock-inf.
PKCα/ MBM
PKCα/ DMSO
PKCα/ DMSO mock-inf.
10 µm

Figure S4. Lack of an effect of MBM on the nuclear localization of NEC-associated cellular factors. HFFs cultivated in 6-well plates were used for infection with HCMV AD169 at a MOI of 0.1 or remained mock-infected, treated with 5 µM of merbromin (MBM, or DMSO as a solvent control) and harvested at 5 d p.i.. Immunofluorescence staining was performed with antibodies against the indicated proteins and representative panels of confocal imaging are given (see scale bar of 10 µm in the panels at the right). Virus-positive cells were visualized by immunostaining with pAb-IE1/IE2. Note, the comparison between MBM-treated HCMV-positive cells (each upper series of pictures), DMSO-treated HCMV-positive cells (each middle series of pictures) and DMSO-treated mock-infected cells (each lower series of pictures).

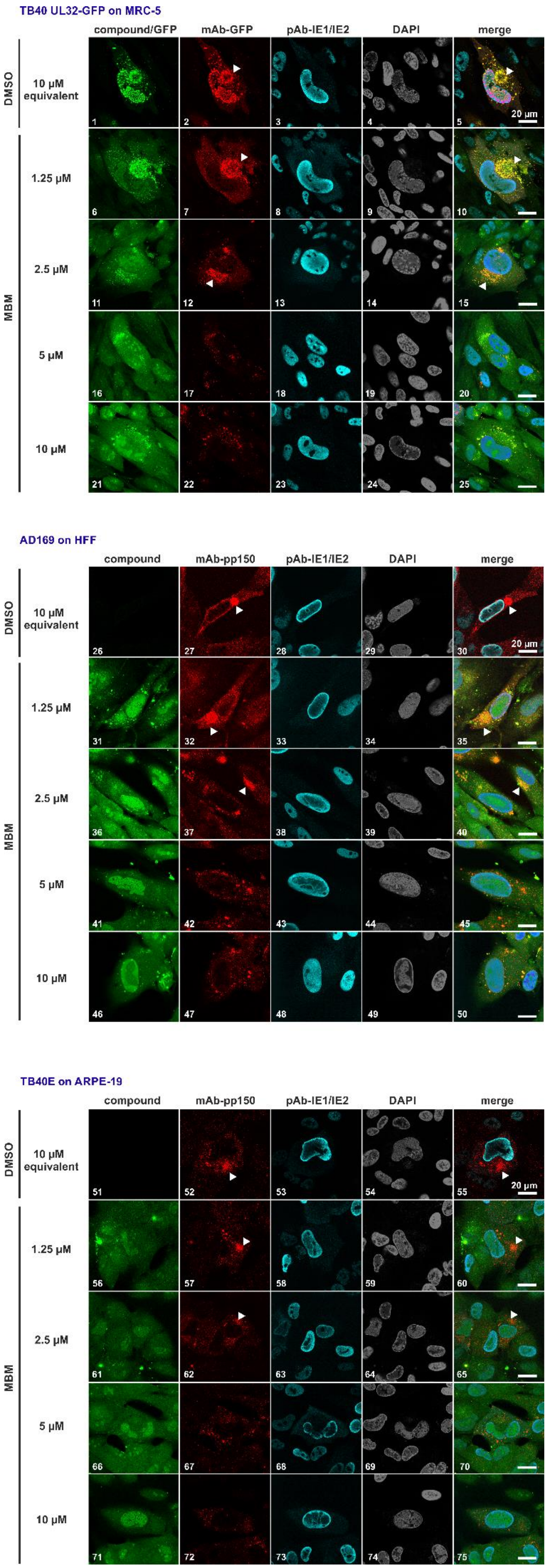
TB40 UL32-GFP on MRC-5
compound/GFP
mAb-GFP
pAb-IE1/IE2
DAPI
merge
DMSO
10 µM equivalent
MBM
1.25 µM
2.5 µM
5 µM
10 µM
20 µm
AD169 on HFF
compound
mAb-pp150
pAb-IE1/IE2
DAPI
merge
DMSO
10 µM equivalent
MBM
1.25 µM
2.5 µM
5 µM
10 µM
20 µm
TB40E on ARPE-19
compound
mAb-pp150
pAb-IE1/IE2
DAPI
merge
DMSO
10 µM equivalent
MBM
1.25 µM
2.5 µM
5 µM
10 µM
20 µm

Figure S5. Confocal microscopic evaluation of pp150-positive cVAC formation in HCMV-infected cells in the absence or presence of MBM treatment. Three different human cell types, namely MRC-5, HFF and ARPE-19, were cultivated in 6-well plates on cover slips and used for the infection with three different strains of HCMV (MOI ≤0.1) and fixed at 5 d p.i.. Indirect immunofluorescence staining was performed for viral pp150 (pUL32), representing a marker of viral cVAC formation, and IE1/IE2 proteins, used as an infected-cell control. Nuclear counterstaining was additionally performed (DAPI) and the presence of the drug (MBM; 1.25-10 µM as indicated) was visualized by its autofluorescence (TB40 UL32-GFP) or by indirect immunofluorescence staining (AD169 and TB40E). White triangles indicate the formation of fully-shaped cVACs. MBM, merbromin; DMSO, solvent control without MBM; scale bar marks 20 µm.
